# Breast and axillary marking in the neoadjuvant setting: survey results from experts of the Brazilian society of mastology

**DOI:** 10.3389/fonc.2024.1393417

**Published:** 2024-10-09

**Authors:** Henrique Lima Couto, Augusto Tufi Hassan, Dalton Ivan Steinmacher, Eduardo Carvalho Pessoa, Eduardo Camargo Millen, Felipe Zerwes, Francisco Pimentel Cavalcante, Giuliano Tosello, Guilherme Novita, Gustavo Machado Badan, José Luis Esteves Francisco, Leonardo Ribeiro Soares, Lucas Roskamp Budel, Luciano Fernandes Chala, Raquel Civolani Marques Fernandes, Ruffo Freitas-Junior, Vilmar Marques de Oliveira, Vinicius Milani Budel, André Mattar

**Affiliations:** ^1^ Brazilian Society of Mastology, Rio de Janeiro, RJ, Brazil; ^2^ Redimama - Redimasto, Belo Horizonte, MG, Brazil; ^3^ Grupo Oncoclínicas, Salvador, BA, Brazil; ^4^ UniCesumar, Maringá, PR, Brazil; ^5^ Botucatu Medical School (UNESP), Botucatu, SP, Brazil; ^6^ Américas Oncologia, Rio de Janeiro, RJ, Brazil; ^7^ Pontifical Catholic University of Rio Grande do Sul, Porto Alegre, RS, Brazil; ^8^ Fortaleza General Hospital, Fortaleza, CE, Brazil; ^9^ Instituto do Câncer Oeste Paulista, Presidente Prudente, SP, Brazil; ^10^ Grupo Oncoclínicas, São Paulo, SP, Brazil; ^11^ Santa Casa de São Paulo, São Paulo, SP, Brazil; ^12^ São José do Rio Preto Medical School, São José do Rio Preto, SP, Brazil; ^13^ Federal University of Goiás, Goiânia, GO, Brazil; ^14^ Federal University of Paraná, Curitiba, PR, Brazil; ^15^ Grupo Fleury Medicina e Saúde, São Paulo, SP, Brazil; ^16^ Laboratório de Anatomia Patológica e Biologia Molecular da Rede Américas, São Paulo, SP, Brazil; ^17^ Hospital da Mulher, São Paulo, SP, Brazil

**Keywords:** breast neoplasm, consensus development conferences, neoadjuvant therapy, sentinel lymph node, breast tumor markers

## Abstract

**Introduction/objectives:**

The precise location of the tumor site is essential for the success of surgical treatment. Neoadjuvant chemotherapy (NAC) is a challenge for preoperative tumor and node localization. Thus, the knowledge and attitudes of the affiliated members of the Brazilian Society of Mastology (SBM) regarding breast and axilla marking were evaluated and a consensus regarding management and treatment was reached.

**Methods:**

This was an online survey conducted between June and December 2022. All 1,742 active mastologists affiliated to the SBM were invited anonymously. The online form contained 28 objective questions, of which 22 were formulated on a Likert scale. These questions addressed relevant aspects related to breast and axilla marking in the neoadjuvant setting. Responses that reached 70% agreement were considered consensual. Statistical analysis was performed using the SPSS program version 26.0. *Post hoc* analysis was performed when appropriate and the significance level was set at p < 0.05. Polychoric regression analyses were conducted using `VGAM` package

**Results:**

In total, 468 mastologists answered the questionnaire (26.8%), with a predominance of professionals aged between 40–49 years (32.1%). Most professionals were board-certified (84,8%). The indication of tumor marking in the breast prior to NAC was consensual (96.4%) and the metal clip was the preferred method (69.7%). There was no consensus regarding the indication of pre-NAC histologically positive lymph node marking (49.8% disagree and 42.8% agree). However, there was consensus that the clinical and imaging evaluation was insufficient for staging the axilla as N1 (71.6%). The contraindication of breast and node marking in T4b tumors (71.2%) was consensual. There was consensus on the indication of sentinel lymph node biopsy (SLNB) for initially cN1 (92.3%) or cN2 (72.7%) tumors that became cN0 after NAC, with 67.5% opting for dual staining with technetium and patent blue. When <3 lymph nodes were retrieved 41.0% of mastologists performed axillary lymphadenectomy. Among the 28 questions, consensus was reached on only 11 (39.3%).

**Conclusion:**

The indication of pre-NAC breast marking is consensual among Brazilian mastologists, although axillary nodal marking is not. There is a great divergence of attitudes among Brazilian surgeons in relation to the many issues related to pre-NAC breast and axilla marking.

## 1 Introduction

Surgical treatment is the therapeutic foundation for most breast-cancer cases. In this context, accurate tumor site localization is crucial for achieving clear margins and surgical treatment success, thereby decreasing the reoperation rates and local recurrences (1, 2). Despite recent meta-analysis findings, the rates of positive margins and reoperations in conservative surgeries for non-palpable tumors were 17% and 16%, respective (2). Consequently, various tumor-marking techniques have been established and refined recently, notably including radioactive seed localization (RSL) using iodine-125 and marking with activated charcoal. Despite limitations in accessibility and financial cost, these techniques decrease the rates of positive margins and reoperation to approximately 7–12% (2, 3).

Administering neoadjuvant chemotherapy (NAC) presents another challenge for preoperative tumor localization owing to varying locoregional response patterns and limitations associated with imaging techniques (4, 5). In this scenario, uncertain tumor site localization and insufficient resection may lead to higher recurrence rates in patients undergoing NAC (21.4% over 15 years) compared to those of patients undergoing adjuvant chemotherapy (15.9%) (6). In clinical practice, these challenges lead to significant variation in breast and axilla clipping indications, including the primary population, most appropriate timing, and method to be used (7, 8).

In recent years, the potential to de-escalate surgical treatment following NAC has broadened the debate on tumor localization to include axillary lymph nodes (9). In this scenario, cases of patients with initially involved axilla (cN+) who achieved cN0 status post-treatment are noteworthy, as sentinel lymph node biopsy (SLNB) in these cases has a relatively high false-negative rate (FNR) of 12–14% (10, 11) and there is not an international consensus on the surgical approach (12, 13). Despite the FNR not leading to worse clinical outcomes after a 10-year follow-up, various studies and international consensus have started to recommend the removal of ≥3 doubly marked lymph nodes and/or clipping of the affected lymph node to achieve a FNR <10% (8, 9, 14).

In Brazil, mastology is a well-established specialty. These professionals are not just breast surgeons; their training encompasses diagnostic imaging, percutaneous minimally invasive procedures including preoperative markings, oncological surgical procedures, breast reconstructions, systemic treatment, and monitoring of high-risk patients (15). A survey among Brazilian mastologists in 2020 found that approximately 13% of them recommended axillary lymph node dissection (ALND) for all post-NAC cases, and only 18% performed SLNB for patients with cN0 tumors. Additionally, there were various differing approaches regarding preoperative axillary assessment, lymph node marking methods, and management of ypN+ (16).

Considering the debates surrounding tumor marking and axillary management, particularly in the NAC setting, we conducted a survey among Brazilian mastologists to understand their clinical practices and the challenges they face. Ultimately, the consensus recommendations and key areas of disagreement among the Brazilian Society of Mastology (SBM) members who responded to the survey were highlighted.

## 2 Methods

A panel of experts including members of the SBM, a radiologist and a pathologist made a questionnaire (supplementals), and an online survey was conducted between June and December 2022.

All 1,742 active SBM members were invited to participate anonymously. All fully completed questionnaires were included.

The form consisted of 28 multiple-choice questions and was created using Google Forms. Each question offered five response options, with 22 structured on a Likert scale ranging from ‘strongly disagree’ to ‘strongly agree,’ including ‘disagree,’ ‘neutral,’ and ‘agree somewhat`. The issues addressed various important aspects related to breast and axillary clipping, primarily in the neoadjuvant treatment setting. We adhered to the guidelines of the American Association for Public Opinion Research in the creation of the survey and the assessment of response (17).

The initial section of the survey focused on the demographic information of the respondents, encompassing gender, board certification in mastology by the SBM (yes or no), geographic region, and nature of professional affiliation (public or private institution). The second section of the questionnaire focused on tumor and lymph node marking, covering clinical indications, preferred methods, the application of axillary ultrasound, and the availability of a breast-imaging specialist. Finally, the third section of the form addressed surgical approach post-NAC, including indications for SLNB or ALND, surgical management without prior clipping, and the use of charcoal as a marking method. To exclude atypical cases, we assumed that the questions pertain to patients in good general health, without an increased risk for bleeding and with a life expectancy of >10 years. Responses that achieved 70% agreement were deemed consensual.

The study was designed as a consensus development conference, a methodological approach aimed at synthesizing expert opinions on complex issues where the evidence may be incomplete or contradictory. This type of study typically relies on structured group processes to generate a collective agreement or decision among a panel of experts (18).

### 2.1 Sample size

To calculate the required sample size, we considered the entire population of active members within the SBM, totaling 1,742 individuals. We adopted the statistical method proposed by Lwanga and Lemeshow (1991) (19). To ensure robust statistical power and the generalizability of our findings, we set the parameters to a 95% confidence interval with a 5% margin of error. We also adopted a conservative estimate of the proportion at 50% to enhance the sample’s representativeness across various subgroups within the population. This approach resulted in a calculated sample size of 315 participants, which is statistically adequate to accurately reflect the characteristics of the target population and to guarantee the reliability of the study outcomes.

### 2.2 Statistical analysis

Statistical analysis was conducted using the Statistical Package for the Social Sciences, version 26.0 (IBM Corporation, Armonk, NY, USA). The answers were characterized using absolute (n) and relative (%) frequencies. A *post-hoc* analysis was conducted when relevant (20). A significance level of 5% (p < 0.05) was adopted for all analyses.

Polychoric multivariable regression analyses were conducted encompassing 22 different Likert response variables. The predictors included mammography habilitation, board-certification by the Brazilian Society of Mastology (TEMa), graduation (years), if place of residence was a state capital, gender or sex, and age (years). For each response variable, were extracted the coefficients (B), standard errors, z-values, and p-values for each predictor variable to determine the significance and strength of the predictors. `VGAM` package in the R statistical environment was used.

### 2.3 Ethical issues

The study procedures were conducted in compliance with current Brazilian legislation and the Helsinki Convention. The SBM review board approved the study protocol and publication of the results. Returning a completed questionnaire implied agreement to participate in the study and the consent to publish was obtained from all participants.

## 3 Results

The survey received responses from 468 mastologists (26.8%), predominantly aged 40–49 (32.1%), male (50.4%), and residing in state capitals (63.9%). Most professionals were board-certified as mastologists by SBM (84.8%), with 87.7% certified for >5 years, 119 (25.4%) had mammography habilitation (board certified in mammography), therefore qualified to report mammograms (Brazilian regulation). The other technical and demographic characteristics of the analyzed population are presented in **Table 1**.

**Table 1:** Characterization of the demographic profiles.

	N	%
Age group (years)		
<40	123	26.3
40–49	150	32.1
50–59	120	25.6
≥60	75	16
Sex		
Female	232	49.6
Male	236	50.4
Geographical region of residence/work		
Middle West	42	9
North East	83	17.7
North	14	3
South East	251	53.6
South	78	16.7
Type of institution		
Private	235	50.2
Public	90	19.2
Public and private	143	30.6
Place of residence in capital		
No	169	36.1
Yes	299	63.9
TEMa^**^		
No	71	15.2
Yes	397	84.8
TEMa^**^ group (years)		
<5	49	12.3
5–20	190	47.9
>20	158	39.8
Graduation to TEMa^**^ group (years)		
<5	85	22.1
5–20	282	73.2
>20	18	4.7
Mammography habilitation***		
No	349	74.6
Yes	119	25.4
Mammography habilitation group (years)		
<5	22	18.5
5–20	65	54.6
>20	32	26.9
Graduation to mammography habilitation group (years)		
<5	15	12.6
5–20	96	80.7
>20	8	6.7

*n, absolute frequency; %, relative frequency.

**TEMa, board-certification by the Brazilian Society of Mastology.

***Board-certification and license by Brazilian Medical Association to analyze and report mammograms and be medically responsible for mammographers sites.

Among the practical conditions of the participating mastologists, only 15 (3.2%) reported a complete unavailability of breast marking (**Supplementary Tables 1**, **2**). Most mastologists reported the availability of breast marking with a metallic clip (83.1%), followed by skin tattoo (40.0%), activated charcoal (12.8%), and RSL with iodine-125 (7.5%). Regarding axillary marking, 160 (34.2%) mastologists reported unavailability in clinical practice, predominantly among professionals working in the public health system (**Supplementary Table 3**). Among the available methods, metallic clips (53.0%) and activated charcoal (17.1%) were more commonly accessible (**Supplementary Table 1**).

Out of the 28 questions, consensus was reached on only 11 (39.3%). Approximately 96% of experts agreed on the recommendation for pre-NAC breast clipping (consensus), with a preference for metallic clips (69.7%). There was no consensus around the best time for clipping the breast before NAC, but the most common preference was after obtaining immunohistochemistry results (35.7%; **Table 2**). If available, ultrasound-visible clips were consensual preferred (86.5%), especially among southeast region mastologists (p = 0.001; **Supplementary Table 4**). The availability of a trained breast interventionalist was consensual considered important for pre-NAC breast and axilla marking by 93.3% and 79.1% of respondents, respectively. Furthermore, 93.3% of mastologists consensual considered issues related to techniques and materials as relevant (**Table 2**).

**Table 2:** Summary of the consensus of all professionals.

Questions	Disagreement n (%)	Agreement n (%)	Consensus item on subjective questions	Consensus reached*
1) Breast clipping before NAC is indicated?	11 (2.4)	451 (96.4)	–	Yes
2) Breast clipping before NAC is indicated only when BCS is possible?	108 (23.1)	352 (75.2)	–	Yes
3) In T4b tumors clipping (breast or axilla) before NAC in unnecessary	119 (25.4)	333 (71.2)	–	Yes
4) Suspicious lymph node clipping is necessary before NAC?	285 (60.9)	141 (30.1)	–	No
5) Positive lymph node clipping is necessary before NAC?	233 (49.8)	200 (42.8)	–	No
6) Which method is available for clipping the breast in your routine?	79 (16.9)	389 (83.1)	Metallic clip	Yes
7) Which method is available for clipping the axilla in your routine?	220 (47.0)	248 (53.0)	Metallic clip	No
8) What is your clipping preference?	142 (30.3)	326 (69.7)	Metallic clip	No
9) If the clip is visible by US this would be my preference	51 (10.9)	405 (86.5)	–	Yes
10) Is image or clinical axillary invasion sufficient?	335 (71.6)	126 (26.9)	–	No
11) For breast/axilla clipping the material and technics are important issues	19 (4)	437 (93.3)	–	Yes
12) Available of breast image specialist is considered to breast clipping	26 (5.5)	432 (92.3)	–	Yes
13) Available of breast image specialist is considered to axilla clipping	62 (13.3)	370 (79.1)	–	Yes
14) When is the best moment to clip the breast?	301 (64.3)	167 (35.7)	After the result of the immunohistochemical panel	No
15) What is your opinion about training in lymph node clipping?	99 (33.1)	266 (57.9)	Important/essential	No
16) Have you ever clipped a lymph node?	187 (40.0)	281 (60.0)	Never	No
17) The type of neoadjuvant treatment does not interfere with my clipping	204 (43.6)	242 (51.7)	–	No
18) The cancer subtype changes my clipping recommendation	181 (38.7)	261 (55.7)	–	No
19) SLNB is possible after cN1 and complete clinical/image response	29 (6.2)	432 (92.3)	–	Yes
20) SLNB is possible after cN2 and complete clinical/image response	116 (24.8)	340 (72.7)	–	Yes
21) Axillary clipping is not necessary when use both technetium and blue dye	125 (26.7)	307 (65.6)	–	No
22) SLNB with double marker should be my choice	118 (25.3)	316 (67.5)	–	No
23) Without double marker, axillary clipping is a good option	126 (27)	291 (62.2)	–	No
24) Axillary clearance is my preference when only blue dye or technetium is available	363 (77.6)	89 (19.0)	–	Yes
25) Axillary clearance is my preference when at least three (3) nodes are found	249 (53.2)	192 (41.0)	–	No
26) When the breast is not clipped and a complete response is found I do prefer mastectomy	205 (43.8)	249 (53.2)	–	No
27) Activated coal in the breast do not affect pathological report	136 (29.0)	145 (31.0)	–	No
28) Activated coal in the axilla do not affect pathological report	145 (31.0)	150 (32.0)	–	No

n, absolute frequency; %, relative frequency; values <100% correspond to responses marked as “neutral”.

*Responses that reached 70% agreement.

BCS, breast conserving surgery; NAC, neoadjuvant chemotherapy; SLNB, sentinel lymph node biopsy; US, ultrasound.

There was no consensus regarding the recommendation for marking a histologically positive lymph node before NAC, with 49.8% disagreeing and 42.8% agreeing (**Table 2**). However, clinical and imaging assessments were consensual inadequate for staging the axilla as N1 (71.6%). The expert panel strongly agreed in marking of the breast tumor before NAC (96,4% **Table 2**), although it was contraindicated in T4b tumors (71.2%; **Table 2**). Additionally, 65.6% considered axillary clipping unnecessary when dual marking with technetium and patent blue was available (**Table 2**).

There was consensus on recommending SLNB for initially cN1 (92.3%) or cN2 (72.7%) tumors that became cN0 after NAC, with 67.5% favoring dual marking with technetium and patent blue. There was also consensus in not indicating axillary lymphadenectomy (77.6%) after NAC when double marking with technetium and patent blue for SLNB was not available. The preference for axillary lymphadenectomy was 19.0% without dual marking, and 41.0% when fewer than three lymph nodes were detected in SLNB (**Table 2**). Among the practical issues with a statistically significant difference, mastologists with board certification tended to adopt more conservative approaches compared to those without it.

The multivariable analysis results statistically significant for each predictor and Likert response are outlined in **Table 3**. Breast clipping before NAC is more indicated by mastologists that live in state capitals (p=0.022). Board certified mastologists (TEMa) indicate breast clipping before NAC only when BCS is possible (p=0.006), consider clipping breast or axilla before NAC unnecessary for T4b tumors (p=0.093); do not consider lymph node clipping before NAC necessary (p=0.019) and consider SLNB is possible after cN1 (p=0.037) and cN2 (p=0.014) and complete clinical/image response. Older Mastologists consider lymph node clipping is necessary before NAC (p=0.041), SLNB with double marker should be their choice after NAC (p=0.045) and axillary clearance is their preference when only blue dye or technetium is available for SLNB after NAC (p=0.006) while younger mastologists consider SLNB is possible after cN1 (p=0.006) and cN2 (p=0.045) and complete clinical/image response. The entire multivariable analysis is available in the supplementals.

**Table 3:** Multivariable analysis results statistically significant.

Dependent variable	B	SD	z	p
1) Breast clipping before NAC is indicated?				
Place of residence in capital	0,456	0,199	2,292	0,022
2) Breast clipping before NAC is indicated only when BCS is possible?				
TEMa	0,722	0,262	2,758	0,006
Graduation (Year)	-0,100	0,040	-2,495	0,013
3) In T4b tumors, clipping (breast or axilla) before NAC in unnecessary.				
TEMa	0,413	0,246	1,681	0,093
4) Suspicious lymph node clipping is necessary before NAC?				
Mammography habilitation	-0,531	0,199	-2,673	0,008
5) Positive lymph node clipping is necessary before NAC?				
TEMa	-0,566	0,241	-2,346	0,019
Age (years)	0,077	0,037	2,048	0,041
9) If the clip is visible by US this would be my preference				
Mammography habilitation	0,583	0,244	2,392	0,017
10) Image or clinical axillary invasion is sufficient?				
Gender or Sex	0,684	0,201	3,403	0,001
11) For breast/axilla clipping the material and techniques are important issues				
Gender or Sex	-0,574	0,224	-2,561	0,010
13) Available of breast image specialist is considered to axilla clipping?				
Gender or Sex	-0,669	0,212	-3,152	0,002
19) SLNB is possible after cN1 and complete clinical/image response				
TEMa	0,609	0,292	2,083	0,037
Graduation (Year)	0,094	0,042	2,245	0,025
Age (years)	-0,123	0,044	-2,767	0,006
20) SLNB is possible after cN2 and complete clinical/image response				
TEMa	-0,632	0,257	-2,457	0,014
Graduation (Year)	0,081	0,035	2,322	0,020
Age (years)	-0,073	0,036	-2,008	0,045
22) SLNB with double marker should be my choice				
Graduation (Year)	-0,083	0,037	-2,273	0,023
Age (years)	0,106	0,038	2,770	0,006
24) Axillary clearance is my preference when only blue dye or technetium is available.				
Age (years)	0,079	0,038	2,071	0,038
25) Axillary clearance is my preference when at least 3 nodes are found				
Gender or Sex	-0,387	0,191	-2,025	0,043
28) Activated coal in the axilla do not affect pathological report.				
Gender or Sex	0,377	0,190	1,979	0,045

NAC, neoadjuvant chemotherapy; BCS, breast conservative surgery; US, ultrasound; SLNB, sentinel node biopsy; TEMa, board certification from Brazilian Society of Mastology.

## 4 Discussion

### 4.1 Participants: profiles and experiences

The profiles of the examined mastologists surveyed aligned with previous studies and indicated the state of the specialty in Brazil, which is characterized by sex equality, high technical proficiency, and a predominance of professionals working in major urban centers (16, 21). Since most of them are certified, we can assume that they have a high level of experience. Breast clipping before NAC is more indicated by mastologists that live in state capitals and most breast specialists had never performed axillary clipping (60% **Table 2**), which hindered access to the procedure and surgical training across the country that could underscore the significance of the current study. This situation probably also occurs in many other developing countries. The use of TAD significantly reduces the false negative rate, reaching levels as low as 1.4% (22). Despite this, we know that the removal of 3 lymph nodes and the use of dual marking can reduce the false negative rate to acceptable levels of about 10% (10, 11, 23) and that could be the explanation for not adopting axillary positive lymph node marking not only in Brazil, but also in other countries.

In clinical practice, the metallic clip remains the preferred and most available method. This is likely because it is safe, low cost, user-friendly, and can be inserted during the biopsy (24). The preference for using metallic clips over other technologies, such as magnetic seeds, might indeed stem primarily from availability, but other factors could explain that. Cost is always a significant factor in medical practice and the approval and regulatory status of different marking agents can also affect their adoption around the world. Although we can directly compare the marking technologies’ costs, there are not literature that addressed specifically cost-effectiveness. Magnetic seeds are not approved for pre-NAC marking in Brazil for example. For sure the amount and quality of clinical evidence supporting the use of new technologies affect their adoption and there is extensive evidence supporting the efficacy and safety of metallic clips compared to newer options (25).

However, a significant number of breast specialists (40%) preferred or relied on skin tattoo, probably related to cost, availability and lack of training in other approaches. This method has limitations in determining lesion depth and can lead to larger tissue resections and up to a 33% failure rate in post-NAC localization (26, 27). Conversely, the limited availability of iodine-125 seeds among respondents was noted. While it was the preferred method for 21% of mastologists, <8% had it available in clinical practice and this could have interfered in the results of the study. As previously mentioned, its use for axillary and breast marking is not regular approved in Brazil. Some important alternatives have been tested as feasible method of breast tumor localization surgery (28, 29) and may provide additional benefit over wire localization from advanced scheduling and improved patient and surgical flow.

The use of a clip to mark the lymph node adds costs and requires an additional procedure to preoperative localization with a wire or radioactive seed, which is uncomfortable for patients. In this context, tattooing positive lymph nodes with charcoal raises as a low‐cost alternative technique (30, 31) specially for low-middle income countries. Our data showed a contrast 17% availability yet only 4% of respondents preferred it, possibly owing to its novelty and the perception that it could affect the pathological report.

It might be surprising 29.9% of respondents did not feel that a trained breast interventionalist would be important for pre-NAC axillary marking. Some doctors might not be fully aware of the complexities involved in pre-NAC axillary marking. This is a technique that requires a high level of precision and specialized knowledge, particularly in breast imaging and image-guided interventions. Also, there might be significant variations in clinical practice between different regions or institutions. In some places, it might not be common to have a specialist dedicated exclusively to breast procedures, which could influence opinions on the need for such specialization. Some professionals might believe that radiologists with generic training are adequately capable of performing axillary marking without the need for a breast imaging specialist, and lastly the availability of resources, including financial and human, can affect the decision to employ specialists in specific areas. This is probably a current discussion in all countries.

The results of multiple regression analyses demonstrated the great divergence of attitudes among mastologists` profile. Board certified mastologists seems to be more concerned about costs and more progressive in de-escalation of axillary surgery. They mostly indicate breast clipping before NAC only when breast conservative surgery (BCS) is possible, do not consider lymph node clipping before NAC necessary and consider SLNB is possible after cN1 and cN2 and complete clinical/image response. Older mastologists seems to be more conservative in de-escalation of axillary surgery. They consider lymph node clipping is necessary before NAC, SLNB with double marker should be their choice after NAC and axillary clearance is their preference when only blue dye or technetium is available for SLNB after NAC.

### 4.2 Breast marking

Breast marking is a crucial step for the success of surgical treatment, especially for non-palpable lesions and post-NAC conditions. Despite being a safe and straightforward procedure, technical failures in breast marking can lead to improper resections, positive margins, and increased local recurrence rates (1, 2). Thus, most participants value the availability of a trained breast interventionalist. Despite the emphasis on expertise, it should not prevent general practitioners, especially in low-middle income countries, from performing tumor marking, thereby increasing availability and access to the procedure.

Considering the visualization method there was unanimous preference for ultrasound-guided marking. This preference could stem from several factors as availability and cost (29). Ultrasound is typically less expensive than other imaging methods like mammography and it is a real time exam, easy to use and it also does not involve ionizing radiation. While preferences can guide general practices, specific clinical situations often dictate the methods and technologies required, regardless of general preferences or resource considerations. In cases where tumors are not seen by ultrasound, stereotactic methods remain the only option.

The preference for breast marking after immunohistochemistry is justified once the decision of NAC is most of times after its results and there is the potential to omit the procedure, saving financial resources, in luminal tumors, which are typically treated with upfront surgery rather than NAC (32). Certainly, this situation may repeat in many low-middle income countries.

Finally, given the locally advanced nature of T4b tumors, the consensus against breast clipping reflects a preference for radical modified mastectomy and the understanding that the procedure will not be required for this surgery. This advanced stage reduces the utility of breast clipping once a mastectomy probably will be done.

### 4.3 Axilla marking

Preoperative assessment of axillary status can impact surgical strategy, systemic treatment, and the recommendation for adjuvant radiotherapy (32). Despite advancements in clinical examination and imaging of the axilla, significant rates of false positives and negatives persist in preoperative assessment (33). After NAC, detecting residual disease through imaging methods remains imprecise owing to both technical and human factors (34). Collectively, these findings support pre-NAC biopsy of suspicious axillary nodes and there was consensus on this.

In instances of confirmed positive axilla, the absence of consensus on axillary clipping is attributed to differing opinions on the safety and effectiveness of SLNB in this group (16, 35). Despite a slight increase in FNR with unmarked SLNB (10, 11), many experts believe that this will not lead to decreased overall survival or increased axillary recurrence (36), potentially rendering axillary marking unnecessary. This is a hot debate everywhere, not only in Brazil. Additionally, FNR can be decreased through dual marking, removal of three or more lymph nodes, and/or the addition of immunohistochemistry (10, 11, 23). If indicated, a recent study compared targeted axillary dissection using three different localization markers (clip + iodine seed, magnetic seed, and carbon suspension) and found no significant difference in safety and efficacy outcomes (37).

### 4.4 Axillary management after NAC

Progress made in SLNB after NAC has followed the same pathway as upfront SLNB in that no randomized studies on oncological safety have been conducted up to the present time, irrespective of initial axillary node status. In patients with initially negative axilla (cN0) who receive NAC, FNRs are generally acceptable (≤10%) (38), and, indeed, data from non-randomized studies have shown low rates of axillary recurrence (39). Conversely, patients with clinically positive axilla (cN1/2) prior to treatment and who experience clinical complete response represent a more challenging group. In addition to the lack of randomized studies assessing clinical outcomes, overall FNRs are considered high, possibly impacting on local control and important prognostic information (10, 22, 23, 40). The high agreement on performing SLNB post-NAC in cN+ women who became cN0 reflects the confidence of Brazilian mastologists in the surgical de-escalation process of the axilla and it seems to be a world tendency (41). In 2020, agreement for initially cN1 cases was just 42% (16); it increased to 92% in the current series. In this context, the significance of dual marking and the removal of ≥3 lymph nodes were emphasized, following the methodology employed by pivotal studies in the field (10, 11). In the absence of these criteria, axillary clipping may be a viable option to keep the FNR <10% (12, 13). It is worthy to notice that board certified mastologists are more likely to adopt conservative management of axilla, the consensus made by the American Society of Breast Surgeons in 2022 goes in the same direction (42). This emphasizes the importance of educational programs and board certification.

More recently, there has been an increasing tendency to omit axillary dissection in patients with a positive SLNB (ypN+) following NAC, in cases of isolated tumors cells (ITCs) and micro metastasis (21, 43, 44). Some prospective trials are still evaluating this topic (45–48). Since we do not have a definitive answer, the tendency among professional continues to be a complete axillary clearance or radiotherapy in this situation (21, 42).

### 4.5 Limitations

This study had some limitations. Although, it had a cross-sectional design and low rate of participation of Brazilian mastologists, the calculated sample size of 315 was sufficiently exceeded (468) and our results are still valuable. Our approach followed established guidelines and practices for conducting and reporting consensus development studies, ensuring that the findings are both robust and directly applicable to clinical practice.

Despite sample exceeded the calculated sample size, non-responders can differ from responders (R), even in other aspects than responding, resulting in non-responder bias (49–54). It was not possible to directly address the non-responder bias once the survey was anonymous. Nevertheless, the responder cohort was not very distinct to SBM members features in distribution by age: 59.7%(SBM) x 58.4%(R) < 50, 40.3%(SBM) x 41,6%(R) >50; and by geographical region of residence/work. Although, there were differences by gender: 43%(SBM) x 50.4%(R) male and 57%(SBM) x 49.6%(R) female; and by board certification 64,9%(SBM) x 84.8%(R).

However, this rate aligns with other surveys conducted by the SBM and likely reflects the experiences and views of other Brazilian professionals. In contrast, this represents the largest survey on breast and axilla marking conducted in a low- and middle-income country, which could enhance the understanding of global regional differences and improve clinical practice management. When a survey claims to enhance understanding of global and regional practices based solely on data from one country, it risks providing an incomplete picture. Discussing such a specialized and globally relevant topic, and integrating these elements, would not only address the stated objectives more thoroughly but also enhance the report’s utility for practitioners worldwide. This approach ensures that the survey serves as a valuable resource for understanding and potentially harmonizing practices in axillary management post-NAC across various healthcare landscapes.

### 4.6 Conclusions

Brazilian mastologists reached to consensus concerning the recommendation for pre-NAC breast marking. There is a significant disparity in practices among Brazilian surgeons regarding various aspects of pre-NAC breast and axillary marking, highlighting the need for ongoing educational programs on the subject.

## Data Availability

The original contributions presented in the study are included in the article/**Supplementary Material**. Further inquiries can be directed to the corresponding author.
