# Supplementary material for: Breast and axillary marking in the neoadjuvant setting: survey results from experts of the Brazilian society of mastology
Source: Front Oncol. 2024 Oct 9;14:1393417. doi: 10.3389/fonc.2024.1393417 (PMC11496257; doi:10.3389/fonc.2024.1393417)
Supplement: Supplementary file 1 [file Table1.docx]

**SUPPLEMENTARY MATERIAL**

Summary

[Table S1. Complete panel with all survey responses. 2](#_Toc156056785)

[Table S2. Answers to questions formulated on a Likert scale. 9](#_Toc156056786)

[Table S3. Distribution of variables according to the type of institution of the mastologist. 11](#_Toc156056787)

[Table S4. Distribution of variables according to the geographic location of the mastologist. 15](#_Toc156056788)

[Table S5. Distribution of variables according to the mastologist's age group. 19](#_Toc156056789)

[Table S6. Distribution of variables according to the mastologist's gender group. 23](#_Toc156056790)

[Table S7. Distribution of variables according to the certification board time. 27](#_Toc156056791)

[Table S8. Distribution of variables according to the presence of board certification. 32](#_Toc156056792)

# Table S1. Complete panel with all survey responses.

| **Questions** | **n** | **%** |
| --- | --- | --- |
| **1) Breast clipping before NAC is indicated?** |  |  |
| Totally disagree. | 2 | 0,4 |
| Partially disagree. | 9 | 1,9 |
| Neutral. | 6 | 1,3 |
| Partially agree. | 176 | 37,6 |
| Totally agree. | 275 | 58,8 |
| **2) Breast clipping before NAC is indicated only when BCS is possible?** |  |  |
| Totally disagree. | 56 | 12,0 |
| Partially disagree. | 52 | 11,1 |
| Neutral. | 8 | 1,7 |
| Partially agree. | 131 | 28,0 |
| Totally agree. | 221 | 47,2 |
| **3) In T4b tumors clipping (breast or axilla) before NAC in unnecessary** |  |  |
| Totally disagree. | 39 | 8,3 |
| Partially disagree. | 80 | 17,1 |
| Neutral. | 16 | 3,4 |
| Partially agree. | 151 | 32,3 |
| Totally agree. | 182 | 38,9 |
| **4) Suspicious lymph node clipping is necessary before NAC?** |  |  |
| Totally disagree. | 189 | 40,4 |
| Partially disagree. | 96 | 20,5 |
| Neutral. | 42 | 9,0 |
| Partially agree. | 106 | 22,6 |
| Totally agree. | 35 | 7,5 |
| **5) Positive lymph node clipping is necessary before NAC?** |  |  |
| Totally disagree. | 143 | 30,6 |
| Partially disagree. | 90 | 19,2 |
| Neutral. | 35 | 7,5 |
| Partially agree. | 115 | 24,6 |
| Totally agree. | 85 | 18,2 |
| **6) Which method is available for clipping the breast in your routine?** |  |  |
| None. | 15 | 3,2 |
| Coal. | 60 | 12,8 |
| Metallic clip. | 389 | 83,1 |
| Skin tattoo. | 187 | 40,0 |
| Radioactive seeds. | 35 | 7,5 |
| **7) Which method is available for clipping the axilla in your routine?** | 308 | 65,8 |
| None. | 160 | 34,2 |
| Coal. | 80 | 17,1 |
| Metallic clip. | 248 | 53,0 |
| Skin tattoo. | 40 | 8,5 |
| Radioactive seeds. | 36 | 7,7 |
| **8) What is your clipping preference?** |  |  |
| Coal. | 19 | 4,1 |
| Metallic clip. | 326 | 69,7 |
| None. | 5 | 1,1 |
| Skin tattoo. | 18 | 3,8 |
| Radioactive seeds. | 100 | 21,4 |
| **9) If the clip is visible by US this would be my preference** |  |  |
| Totally disagree. | 27 | 5,8 |
| Partially disagree. | 24 | 5,1 |
| Neutral. | 12 | 2,6 |
| Partially agree. | 95 | 20,3 |
| Totally agree. | 310 | 66,2 |
| **10) Image or clinical axillary invasion is sufficient?** |  |  |
| Totally disagree. | 207 | 44,2 |
| Partially disagree. | 128 | 27,4 |
| Neutral. | 7 | 1,5 |
| Partially agree. | 106 | 22,6 |
| Totally agree. | 20 | 4,3 |
| **11) For breast/axilla clipping the material and technics are important issues** |  |  |
| Totally disagree. | 9 | 1,9 |
| Partially disagree. | 10 | 2,1 |
| Neutral. | 12 | 2,6 |
| Partially agree. | 127 | 27,1 |
| Totally agree. | 310 | 66,2 |
| **12) Available of breast image specialist is considered to breast clipping** |  |  |
| Totally disagree. | 17 | 3,6 |
| Partially disagree. | 9 | 1,9 |
| Neutral. | 10 | 2,1 |
| Partially agree. | 105 | 22,4 |
| Totally agree. | 327 | 69,9 |
| **13) Available of breast image specialist is considered to axilla clipping** |  |  |
| Totally disagree. | 43 | 9,2 |
| Partially disagree. | 19 | 4,1 |
| Neutral. | 36 | 7,7 |
| Partially agree. | 92 | 19,7 |
| Totally agree. | 278 | 59,4 |
| **14) When is the best moment to clip the breast?** |  |  |
| After pathologic hematoxylin-eosin confirmation of malignancy in the biopsy. | 113 | 24,1 |
| After immunohistochemistry prognostic panel result. | 167 | 35,7 |
| During the NAC according to clinical and imaging response. | 47 | 10,0 |
| Breast tumor marking is not needed before NAC | 4 | 0,9 |
| During the biopsy. | 137 | 29,3 |
| **15) What is your opinion about training in lymph node clipping?** |  |  |
| Dispensable. | 99 | 21,2 |
| Optional. | 56 | 12,0 |
| Neutral. | 42 | 9,0 |
| Important | 188 | 40,2 |
| Indispensable | 83 | 17,7 |
| **16) Have you ever clipped a lymph node?** |  |  |
| Never. | 281 | 60,0 |
| 1- 5 procedures. | 104 | 22,2 |
| 5- 10 procedures | 27 | 5,8 |
| 10 – 20 procedures | 18 | 3,8 |
| More than 20 procedures | 38 | 8,1 |
| **17) The type of neoadjuvant treatment does not interfere with my clipping** |  |  |
| Totally disagree. | 121 | 25,9 |
| Partially disagree. | 83 | 17,7 |
| Neutral. | 22 | 4,7 |
| Partially agree. | 115 | 24,6 |
| Totally agree. | 127 | 27,1 |
| **18) The cancer subtype changes my clipping recommendation** |  |  |
| Totally disagree. | 114 | 24,4 |
| Partially disagree. | 67 | 14,3 |
| Neutral. | 26 | 5,6 |
| Partially agree. | 149 | 31,8 |
| Totally agree. | 112 | 23,9 |
| **19) SLNB is possible after cN1 and complete clinical/image response** |  |  |
| Totally disagree. | 15 | 3,2 |
| Partially disagree. | 14 | 3,0 |
| Neutral. | 7 | 1,5 |
| Partially agree. | 73 | 15,6 |
| Totally agree. | 359 | 76,7 |
| **20) SLNB is possible after cN2 and complete clinical/image response** |  |  |
| Totally disagree. | 63 | 13,5 |
| Partially disagree. | 53 | 11,3 |
| Neutral. | 12 | 2,6 |
| Partially agree. | 150 | 32,1 |
| Totally agree. | 190 | 40,6 |
| **21) Axillary clipping is not necessary when use both technetium and blue dye** |  |  |
| Totally disagree. | 52 | 11,1 |
| Partially disagree. | 73 | 15,6 |
| Neutral. | 36 | 7,7 |
| Partially agree. | 130 | 27,8 |
| Totally agree. | 177 | 37,8 |
| **22) SNB with double marker should be my choice** |  |  |
| Totally disagree. | 70 | 15,0 |
| Partially disagree. | 48 | 10,3 |
| Neutral. | 34 | 7,3 |
| Partially agree. | 132 | 28,2 |
| Totally agree. | 184 | 39,3 |
| **23) Without double marker axillary clipping is a good option** |  |  |
| Totally disagree. | 70 | 15,0 |
| Partially disagree. | 56 | 12,0 |
| Neutral. | 51 | 10,9 |
| Partially agree. | 190 | 40,6 |
| Totally agree. | 101 | 21,6 |
| **24) Axillary clearances is my preference when only blue dye or technetium is available** |  |  |
| Totally disagree. | 234 | 50,0 |
| Partially disagree. | 129 | 27,6 |
| Neutral. | 16 | 3,4 |
| Partially agree. | 62 | 13,2 |
| Totally agree. | 27 | 5,8 |
| **25) Axillary clearance is my preference when at least 3 nodes are found** |  |  |
| Totally disagree. | 118 | 25,2 |
| Partially disagree. | 131 | 28,0 |
| Neutral. | 27 | 5,8 |
| Partially agree. | 103 | 22,0 |
| Totally agree. | 89 | 19,0 |
| **26) When the breast is not clipped and a complete response is found I do prefer mastectomy** |  |  |
| Totally disagree. | 71 | 15,2 |
| Partially disagree. | 134 | 28,6 |
| Neutral. | 14 | 3,0 |
| Partially agree. | 153 | 32,7 |
| Totally agree. | 96 | 20,5 |
| **27) Activated coal in the breast does not affect pathological report** |  |  |
| Totally disagree. | 38 | 8,1 |
| Partially disagree. | 98 | 20,9 |
| Neutral. | 187 | 40,0 |
| Partially agree. | 70 | 15,0 |
| Totally agree. | 75 | 16,0 |
| **28) Activated coal in the axilla does not affect pathological report** |  |  |
| Totally disagree. | 46 | 9,8 |
| Partially disagree. | 99 | 21,2 |
| Neutral. | 173 | 37,0 |
| Partially agree. | 81 | 17,3 |
| Totally agree. | 69 | 14,7 |

n, absolute frequence; %, relative frequence.

Legend: BCS, breast conserving surgery; HE, hematoxylin-eosin; NAC, neoadjuvant chemotherapy; SLNB, sentinel lymph node biopsy; US, ultrasound.

# Table S2. Answers to questions formulated on a Likert scale.

|  | Totally disagree. | Partially disagree. | Neutral. | Partially agree. | Totally agree. |
| --- | --- | --- | --- | --- | --- |
| 1) Breast clipping before NAC is indicated? | 2 (0,4) | 9 (1,9) | 6 (1,3) | 176 (37,6) | 275 (58,8) |
| 2) Breast clipping before NAC is indicated only when BCS is possible? | 56 (12,0) | 52 (11,1) | 8 (1,7) | 131 (28,0) | 221 (47,2) |
| 3) In T4b tumors clipping (breast or axilla) before NAC in unnecessary | 39 (8,3) | 80 (17,1) | 16 (3,4) | 151 (32,3) | 182 (38,9) |
| 4) Suspicious lymph node clipping is necessary before NAC? | 189 (40,4) | 96 (20,5) | 42 (9,0) | 106 (22,6) | 35 (7,5) |
| 5) Positive lymph node clipping is necessary before NAC? | 143 (30,6) | 90 (19,2) | 35 (7,5) | 115 (24,6) | 85 (18,2) |
| 9) If the clip is visible by US this would be my preference | 27 (5,8) | 24 (5,1) | 12 (2,6) | 95 (20,3) | 310 (66,2) |
| 10) Image or clinical axillary invasion is sufficient? | 207 (44,2) | 128 (27,4) | 7 (1,5) | 106 (22,6) | 20 (4,3) |
| 11) For breast/axilla clipping the material and technics are important issues | 9 (1,9) | 10 (2,1) | 12 (2,6) | 127 (27,1) | 310 (66,2) |
| 12) Available of breast image specialist is considered to breast clipping | 17 (3,6) | 9 (1,9) | 10 (2,1) | 105 (22,4) | 327 (69,9) |
| 13) Available of breast image specialist is considered to axilla clipping | 43 (9,2) | 19 (4,1) | 36 (7,7) | 92 (19,7) | 278 (59,4) |
| 17) The type of neoadjuvant treatment does not interfere with my clipping | 121 (25,9) | 83 (17,7) | 22 (4,7) | 115 (24,6) | 127 (27,1) |
| 18) The cancer subtype changes my clipping recommendation | 114 (24,4) | 67 (14,3) | 26 (5,6) | 149 (31,8) | 112 (23,9) |
| 19) SNB is possible after cN1 and complete clinical/image response | 15 (3,2) | 14 (3,0) | 7 (1,5) | 73 (15,6) | 359 (76,7) |
| 20) SNB is possible after cN2 and complete clinical/image response | 63 (13,5) | 53 (11,3) | 12 (2,6) | 150 (32,1) | 190 (40,6) |
| 21) Axillary clipping is not necessary when use both technetium and blue dye | 52 (11,1) | 73 (15,6) | 36 (7,7) | 130 (27,8) | 177 (37,8) |
| 22) SNB with double marker should be my choice | 70 (15,0) | 48 (10,3) | 34 (7,3) | 132 (28,2) | 184 (39,3) |
| 23) Without double marker axillary clipping is a good option | 70 (15,0) | 56 (12,0) | 51 (10,9) | 190 (40,6) | 101 (21,6) |
| 24) Axillary clearance is my preference when only blue dye or technetium is available | 234 (50,0) | 129 (27,6) | 16 (3,4) | 62 (13,2) | 27 (5,8) |
| 25) Axillary clearance is my preference when at least 3 nodes are found | 118 (25,2) | 131 (28,0) | 27 (5,8) | 103 (22,0) | 89 (19,0) |
| 26) When the breast is not clipped and a complete response is found I do prefer mastectomy | 71 (15,2) | 134 (28,6) | 14 (3,0) | 153 (32,7) | 96 (20,5) |
| 27) Activated coal in the breast does not affect pathological report | 38 (8,1) | 98 (20,9) | 187 (40,0) | 70 (15,0) | 75 (16,0) |
| 28) Activated coal in the axilla does not affect pathological report | 46 (9,8) | 99 (21,2) | 173 (37,0) | 81 (17,3) | 69 (14,7) |

n, absolute frequence; %, relative frequence.

Legend: BCS, breast conserving surgery; HE, hematoxylin-eosin; NAC, neoadjuvant chemotherapy; SLNB, sentinel lymph node biopsy; US, ultrasound.

# Table S3. Distribution of variables according to the type of institution of the mastologist.

|  | Type of institution | | | *p** |
| --- | --- | --- | --- | --- |
|  | Private | Mixed | Public |  |
| **1) Breast clipping before NAC is indicated?** |  |  |  |  |
| Disagree | 6 (2,6) | 4 (2,8) | 1 (1,1) | 0,451 |
| Neutral | 5 (2,1) | 1 (0,7) | 0 (0,0) |  |
| Agree | 224 (95,3) | 138 (96,5) | 89 (98,9) |  |
| **2) Breast clipping before NAC is indicated only when BCS is possible?** | | |  |  |
| Disagree. | 56 (23,8) | 28 (19,6) | 24 (26,7) | 0,365 |
| Neutral. | 6 (2,6) | 2 (1,4) | 0 (0,0) |  |
| Agree. | 173 (73,6) | 113 (79,0) | 66 (73,3) |  |
| **3) In T4b tumors clipping (breast or axilla) before NAC in unnecessary** | | |  |  |
| Disagree. | 63 (26,8) | 34 (23,8) | 22 (24,4) | 0,523 |
| Neutral. | 11 (4,7) | 3 (2,1) | 2 (2,2) |  |
| Agree. | 161 (68,5) | 106 (74,1) | 66 (73,3) |  |
| **4) Suspicious lymph node clipping is necessary before NAC?** | |  |  |  |
| Disagree. | 144 (61,3) | 88 (61,5) | 53 (58,9) | 0,785 |
| Neutral. | 18 (7,7) | 13 (9,1) | 11 (12,2) |  |
| Agree. | 73 (31,1) | 42 (29,4) | 26 (28,9) |  |
| **5) Positive lymph node clipping is necessary before NAC?** | |  |  |  |
| Disagree. | 117 (49,8) | 74 (51,7) | 42 (46,7) | 0,403 |
| Neutral. | 14 (6,0) | 10 (7,0) | 11 (12,2) |  |
| Agree. | 104 (44,3) | 59 (41,3) | 37 (41,1) |  |
| **6) Which method is available for clipping the breast in your routine?** | | |  |  |
| Disagree. | 7 (3,0) | 7 (4,9) | 1 (1,1) | **0,013** |
| Neutral. | 34 (14,5) | 17 (11,9) | 9 (10,0) |  |
| Agree. | **203 (86,4)≠** | **121 (84,6)≠** | 65 (72,2) |  |
| Skin tattoo. | 75 (31,9) | 61 (42,7) | **51 (56,7)≠** |  |
| Radioactive Seeds. | 24 (10,2) | 7 (4,9) | 4 (4,4) |  |
| **7) Which method is available for clipping the axilla in your routine?** | | |  |  |
| None. | 59 (25,1) | 52 (36,4) | **49 (54,4)≠** | **0,001** |
| Coal. | 43 (18,3) | 22 (15,4) | 15 (16,7) |  |
| Metallic Clip. | **148 (63,0)≠** | **74 (51,7)≠** | 26 (28,9) |  |
| Skin tattoo. | 16 (6,8) | 13 (9,1) | 11 (12,2) |  |
| Radioactive Seeds. | **25 (10,6)≠** | 9 (6,3) | 2 (2,2) |  |
| **8) What is your clipping preference?** |  |  |  |  |
| None. | 3 (1,3) | 2 (1,4) | 0 (0,0) | 0,431 |
| Coal. | 12 (5,1) | 5 (3,5) | 2 (2,2) |  |
| Metallic Clip. | 159 (67,7) | 102 (71,3) | 65 (72,2) |  |
| Skin tattoo. | 5 (2,1) | 8 (5,6) | 5 (5,6) |  |
| Radioactive Seeds. | 56 (23,8) | 26 (18,2) | 18 (20,0) |  |
| **9) If the clip is visible by US this would be my preference** | |  |  |  |
| Disagree. | 27 (11,5) | 14 (9,8) | 10 (11,1) | 0,834 |
| Neutral. | 7 (3,0) | 2 (1,4) | 3 (3,3) |  |
| Agree. | 201 (85,5) | 127 (88,8) | 77 (85,6) |  |
| **10) Image or clinical axillary invasion is sufficient?** | |  |  |  |
| Disagree. | 182 (77,4) | 95 (66,4) | 58 (64,4) | 0,081 |
| Neutral. | 3 (1,3) | 2 (1,4) | 2 (2,2) |  |
| Agree. | 50 (21,3) | 46 (32,2) | 30 (33,3) |  |
| **11) For breast/axilla clipping the material and technics are important issues** | | |  |  |
| Disagree. | 8 (3,4) | 7 (4,9) | 4 (4,4) | 0,919 |
| Neutral. | 5 (2,1) | 4 (2,8) | 3 (3,3) |  |
| Agree. | 222 (94,5) | 132 (92,3) | 83 (92,2) |  |
| **12) Available of breast image specialist is considered to breast clipping** | | |  |  |
| Disagree. | 12 (5,1) | 8 (5,6) | 6 (6,7) | 0,645 |
| Neutral. | 7 (3,0) | 1 (0,7) | 2 (2,2) |  |
| Agree. | 216 (91,9) | 134 (93,7) | 82 (91,1) |  |
| **13) Available of breast image specialist is considered to axilla clipping** | | |  |  |
| Disagree. | 30 (12,8) | 21 (14,7) | 11 (12,2) | 0,441 |
| Neutral. | 22 (9,4) | 6 (4,2) | 8 (8,9) |  |
| Agree. | 183 (77,9) | 116 (81,1) | 71 (78,9) |  |
| **14) When is the best moment to clip the breast?** | |  |  |  |
| After pathologic hematoxylin-eosin confirmation of malignancy in the biopsy. | 47 (20,0) | 33 (23,1) | 33 (36,7) | 0,120 |
| After immunohistochemistry prognostic panel result. | 88 (37,4) | 53 (37,1) | 26 (28,9) |  |
| During the NAC according to clinical and imaging response. | 22 (9,4) | 16 (11,2) | 9 (10,0) |  |
| Breast tumor marking is not needed before NAC | 3 (1,3) | 0 (0,0) | 1 (1,1) |  |
| During the biopsy. | 75 (31,9) | 41 (28,7) | 21 (23,3) |  |
| **15) What is your opinion about training in lymph node clipping?** | | |  |  |
| Dispensable. | 50 (21,3) | 27 (18,9) | 22 (24,4) | 0,650 |
| Optional. | 27 (11,5) | 21 (14,7) | 8 (8,9) |  |
| Neutral. | 21 (8,9) | 9 (6,3) | 12 (13,3) |  |
| Important | 95 (40,4) | 60 (42,0) | 33 (36,7) |  |
| Indispensable | 42 (17,9) | 26 (18,2) | 15 (16,7) |  |
| **16) Have you ever clipped a lymph node?** |  |  |  |  |
| Never. | 129 (54,9) | 89 (62,2) | 63 (70,0) | 0,108 |
| 1- 5 procedures. | 62 (26,4) | 30 (21,0) | 12 (13,3) |  |
| 5- 10 procedures | 18 (7,7) | 4 (2,8) | 5 (5,6) |  |
| 10 – 20 procedures | 7 (3,0) | 8 (5,6) | 3 (3,3) |  |
| More than 20 procedures | 19 (8,1) | 12 (8,4) | 7 (7,8) |  |
| **17) The type of neoadjuvant treatment does not interfere with my clipping** | | |  |  |
| Disagree. | 105 (44,7) | 58 (40,6) | 41 (45,6) | 0,246 |
| Neutral. | 6 (2,6) | 10 (7,0) | 6 (6,7) |  |
| Agree. | 124 (52,8) | 75 (52,4) | 43 (47,8) |  |
| **18) The cancer subtype changes my clipping recommendation** | |  |  |  |
| Disagree. | 99 (42,1) | 48 (33,6) | 34 (37,8) | 0,230 |
| Neutral. | 12 (5,1) | 6 (4,2) | 8 (8,9) |  |
| Agree. | 124 (52,8) | 89 (62,2) | 48 (53,3) |  |
| **19) SNB is possible after cN1 and complete clinical/image response** | | |  |  |
| Disagree. | 13 (5,5) | 7 (4,9) | 9 (10,0) | 0,217 |
| Neutral. | 3 (1,3) | 4 (2,8) | 0 (0,0) |  |
| Agree. | 219 (93,2) | 132 (92,3) | 81 (90,0) |  |
| **20) SNB is possible after cN2 and complete clinical/image response** | | |  |  |
| Disagree. | 55 (23,4) | 34 (23,8) | 27 (30,0) | 0,250 |
| Neutral. | 7 (3,0) | 1 (0,7) | 4 (4,4) |  |
| Agree. | 173 (73,6) | 108 (75,5) | 59 (65,6) |  |
| **21) Axillary clipping is not necessary when use both technetium and blue dye** | | | |  |
| Disagree. | 74 (31,5) | 29 (20,3) | 22 (24,4) | 0,106 |
| Neutral | 15 (6,4) | 11 (7,7) | 10 (11,1) |  |
| Agree | 146 (62,1) | 103 (72,0) | 58 (64,4) |  |
| **22) SNB with double marker should be my choice** | |  |  |  |
| Disagree. | 60 (25,5) | 41 (28,7) | 17 (18,9) | 0,420 |
| Neutral. | 17 (7,2) | 8 (5,6) | 9 (10,0) |  |
| Agree. | 158 (67,2) | 94 (65,7) | 64 (71,1) |  |
| **23) Without double marker axillary clipping is a good option** | |  |  |  |
| Disagree. | 71 (30,2) | 34 (23,8) | 21 (23,3) | 0,081 |
| Neutral. | 26 (11,1) | 10 (7,0) | 15 (16,7) |  |
| Agree. | 138 (58,7) | 99 (69,2) | 54 (60,0) |  |
| **24) Axillary clearance is my preference when only blue dye or technetium is available** | | | |  |
| Disagree. | 186 (79,1) | 111 (77,6) | 66 (73,3) | 0,523 |
| Neutral. | 10 (4,3) | 3 (2,1) | 3 (3,3) |  |
| Agree. | 39 (16,6) | 29 (20,3) | 21 (23,3) |  |
| **25) Axillary clearance is my preference when at least 3 nodes are found** | | |  |  |
| Disagree. | 140 (59,6) | 70 (49,0) | 39 (43,3) | 0,037 |
| Neutral. | 15 (6,4) | 7 (4,9) | 5 (5,6) |  |
| Agree. | 80 (34,0) | 66 (46,2) | 46 (51,1) |  |
| **26) When the breast is not clipped and a complete response is found I do prefer mastectomy** | | | | |
| Disagree. | 108 (46,0) | 54 (37,8) | 43 (47,8) | 0,514 |
| Neutral. | 7 (3,0) | 5 (3,5) | 2 (2,2) |  |
| Agree. | 120 (51,1) | 84 (58,7) | 45 (50,0) |  |
| **27) Activated coal in the breast do not affect pathological report** | | |  |  |
| Disagree. | 69 (29,4) | 46 (32,2) | 21 (23,3) | 0,301 |
| Neutral. | 86 (36,6) | 59 (41,3) | 42 (46,7) |  |
| Agree. | 80 (34,0) | 38 (26,6) | 27 (30,0) |  |
| **28) Activated coal in the axilla do not affect pathological report** | | |  |  |
| Disagree. | 74 (31,5) | 49 (34,3) | 22 (24,4) | 0,428 |
| Neutral. | 82 (34,9) | 51 (35,7) | 40 (44,4) |  |
| Agree. | 79 (33,6) | 43 (30,1) | 28 (31,1) |  |

* Chi-square; ≠Post hoc; n, absolute frequency; %, relative frequency.

Legend: BCS, breast conserving surgery; HE, hematoxylin-eosin; NAC, neoadjuvant chemotherapy; SLNB, sentinel lymph node biopsy; US, ultrasound.

# Table S4. Distribution of variables according to the geographic location of the mastologist.

|  | Geographical region of residence/work | | *p** |
| --- | --- | --- | --- |
|  | Other regions. | Southeast |  |
| **1) Breast clipping before NAC is indicated?** | |  |  |
| Disagree. | 1 (0,5) | 10 (4,0) | **0,054** |
| Neutral. | 2 (0,9) | 4 (1,6) |  |
| Agree. | 214 (98,6) | 237 (94,4) |  |
| **2) Breast clipping before NAC is indicated only when BCS is possible?** | | |  |
| Disagree. | 50 (23,0) | 58 (23,1) | 0,260 |
| Neutral. | 6 (2,8) | 2 (0,8) |  |
| Agree. | 161 (74,2) | 191 (76,1) |  |
| **3) In T4b tumors clipping (breast or axilla) before NAC in unnecessary** | | |  |
| Disagree. | 62 (28,6) | 57 (22,7) | 0,296 |
| Neutral. | 6 (2,8) | 10 (4,0) |  |
| Agree. | 149 (68,7) | 184 (73,3) |  |
| **4) Suspicious lymph node clipping is necessary before NAC?** | |  |  |
| Disagree. | 126 (58,1) | 159 (63,3) | 0,177 |
| Neutral. | 25 (11,5) | 17 (6,8) |  |
| Agree. | 66 (30,4) | 75 (29,9) |  |
| **5) Positive lymph node clipping is necessary before NAC?** | (102 | (131 |  |
| Disagree. | 102 (47,0) | 131 (52,2) | 0,534 |
| Neutral. | 17 (7,8) | 18 (7,2) |  |
| Agree. | 98 (45,2) | 102 (40,6) |  |
| **6) Which method is available for clipping the breast in your routine?** | | |  |
| None. | 5 (2,3) | 10 (4,0) | **0,015** |
| Coal. | 35 (16,1) | 25 (10,0) |  |
| Metallic Clip. | 170 (78,3) | **219 (87,3)≠** |  |
| Skin tattoo. | **105 (48,4)≠** | 82 (32,7) |  |
| Radioactive Seeds. | 19 (8,8) | 16 (6,4) |  |
| **7) Which method is available for clipping the axilla in your routine?** | | |  |
| None. | 74 (34,1) | 86 (34,3) | **0,028** |
| Coal. | 41 (18,9) | 39 (15,5) |  |
| Metallic Clip. | 115 (53,0) | 133 (53,0) |  |
| Skin tattoo. | 19 (8,8) | 21 (8,4) |  |
| Radioactive Seeds. | 23 (10,6)≠ | 13 (5,2) |  |
| **8) What is your clipping preference?** |  |  |  |
| None. | 2 (0,9) | 3 (1,2) | 0,086 |
| Coal. | 11 (5,1) | 8 (3,2) |  |
| Metallic Clip. | 141 (65,0) | 185 (73,7) |  |
| Skin tattoo. | 6 (2,8) | 12 (4,8) |  |
| Radioactive Seeds. | 57 (26,3) | 43 (17,1) |  |
| **9) If the clip is visible by US this would be my preference** | |  |  |
| Disagree. | **35 (16,1)≠** | 16 (6,4) | **0,001** |
| Neutral. | 3 (1,4) | 9 (3,6) |  |
| Agree. | 179 (82,5) | **226 (90,0)≠** |  |
| **10) Image or clinical axillary invasion is sufficient?** | |  |  |
| Disagree. | 153 (70,5) | 182 (72,5) | 0,790 |
| Neutral. | 4 (1,8) | 3 (1,2) |  |
| Agree. | 60 (27,6) | 66 (26,3) |  |
| **11) For breast/axilla clipping the material and technics are important issues** | | |  |
| Disagree. | 7 (3,2) | 12 (4,8) | 0,679 |
| Neutral. | 6 (2,8) | 6 (2,4) |  |
| Agree. | 204 (94,0) | 233 (92,8) |  |
| **12) Available of breast image specialist is considered to breast clipping** | | |  |
| Disagree. | 14 (6,5) | 12 (4,8) | 0,438 |
| Neutral. | 3 (1,4) | 7 (2,8) |  |
| Agree. | 200 (92,2) | 232 (92,4) |  |
| **13) Available of breast image specialist is considered to axilla clipping** | | |  |
| Disagree. | 28 (12,9) | 34 (13,5) | 0,810 |
| Neutral. | 15 (6,9) | 21 (8,4) |  |
| Agree. | 174 (80,2) | 196 (78,1) |  |
| **14) When is the best moment to clip the breast?** | |  |  |
| After pathologic hematoxylin-eosin confirmation of malignancy in the biopsy. | 52 (24,0) | 61 (24,3) | 0,140 |
| After immunohistochemistry prognostic panel result. | 85 (39,2) | 82 (32,7) |  |
| During the NAC according to clinical and imaging response. | 24 (11,1) | 23 (9,2) |  |
| Breast tumor marking is not needed before NAC | 0 (0,0) | 4 (1,6) |  |
| During the biopsy. | 56 (25,8) | 81 (32,3) |  |
| **15) What is your opinion about training in lymph node clipping?** | | |  |
| Dispensable. | 43 (19,8) | 56 (22,3) | 0,643 |
| Optional. | 28 (12,9) | 28 (11,2) |  |
| Neutral. | 18 (8,3) | 24 (9,6) |  |
| Important | 84 (38,7) | 104 (41,4) |  |
| Indispensable | 44 (20,3) | 39 (15,5) |  |
| **16) Have you ever clipped a lymph node?** |  |  |  |
| Never. | 140 (64,5) | 141 (56,2) | 0,356 |
| 1- 5 procedures. | 42 (19,4) | 62 (24,7) |  |
| 5- 10 procedures | 13 (6,0) | 14 (5,6) |  |
| 10 – 20 procedures | 6 (2,8) | 12 (4,8) |  |
| More than 20 procedures | 16 (7,4) | 22 (8,8) |  |
| **17) The type of neoadjuvant treatment does not interfere with my clipping** | | |  |
| Disagree. | 90 (41,5) | 114 (45,4) | 0,667 |
| Neutral. | 10 (4,6) | 12 (4,8) |  |
| Agree. | 117 (53,9) | 125 (49,8) |  |
| **18) The cancer subtype changes my clipping recommendation** | |  |  |
| Disagree. | 86 (39,6) | 95 (37,8) | 0,976 |
| Neutral. | 12 (5,5) | 14 (5,6) |  |
| Agree. | 119 (54,8) | 142 (56,6) |  |
| **19) SNB is possible after cN1 and complete clinical/image response** | | |  |
| Disagree. | 14 (6,5) | 15 (6,0) | 0,654 |
| Neutral. | 2 (0,9) | 5 (2,0) |  |
| Agree. | 201 (92,6) | 231 (92,0) |  |
| **20) SNB is possible after cN2 and complete clinical/image response** | | |  |
| Disagree. | 53 (24,4) | 63 (25,1) | 0,967 |
| Neutral. | 6 (2,8) | 6 (2,4) |  |
| Agree. | 158 (72,8) | 182 (72,5) |  |
| **21) Axillary clipping is not necessary when use both technetium and blue dye** | | |  |
| Disagree. | 64 (29,5) | 61 (24,3) | 0,263 |
| Neutral. | 19 (8,8) | 17 (6,8) |  |
| Agree. | 134 (61,8) | 173 (68,9) |  |
| **22) SNB with double marker should be my choice** | |  |  |
| Disagree. | 53 (24,4) | 65 (25,9) | 0,707 |
| Neutral. | 18 (8,3) | 16 (6,4) |  |
| Agree. | 146 (67,3) | 170 (67,7) |  |
| **23) Without double marker axillary clipping is a good option** | |  |  |
| Disagree. | 57 (26,3) | 69 (27,5) | 0,967 |
| Neutral. | 24 (11,1) | 27 (10,8) |  |
| Agree. | 136 (62,7) | 155 (61,8) |  |
| **24) Axillary clearance is my preference when only blue dye or technetium is available.** | | | |
| Disagree. | **180 (82,9)≠** | 183 (72,9) | **0,002** |
| Neutral. | 10 (4,6) | 6 (2,4) |  |
| Agree. | 27 (12,4) | **62 (24,7)≠** |  |
| **25) Axillary clearance is my preference when at least 3 nodes are found** | | |  |
| Disagree. | 125 (57,6) | 124 (49,4) | 0,178 |
| Neutral. | 10 (4,6) | 17 (6,8) |  |
| Agree. | 82 (37,8) | 110 (43,8) |  |
| **26) When the breast is not clipped and a complete response is found I do prefer mastectomy** | | | |
| Disagree. | 95 (43,8) | 110 (43,8) | 0,967 |
| Neutral. | 6 (2,8) | 8 (3,2) |  |
| Agree. | 116 (53,5) | 133 (53,0) |  |
| **27) Activated coal in the breast do not affect pathological report** | | |  |
| Disagree. | 57 (26,3) | 79 (31,5) | 0,185 |
| Neutral. | 84 (38,7) | 103 (41,0) |  |
| Agree. | 76 (35,0) | 69 (27,5) |  |
| **28) Activated coal in the axilla do not affect pathological report** | | |  |
| Disagree. | 65 (30,0) | 80 (31,9) | 0,558 |
| Neutral. | 77 (35,5) | 96 (38,2) |  |
| Agree. | 75 (34,6) | 75 (29,9) |  |

*Chi-square; ≠Post hoc; n, absolute frequency; %, relative frequency.

Legend: BCS, breast conserving surgery; HE, hematoxylin-eosin; NAC, neoadjuvant chemotherapy; SLNB, sentinel lymph node biopsy; US, ultrasound.

# Table S5. Distribution of variables according to the mastologist's age group.

|  | Age group | | | | *p** |
| --- | --- | --- | --- | --- | --- |
|  | < 40 | 40 to 49 | 50 to 59 | 60 ≥ |  |
| **1) Breast clipping before NAC is indicated?** |  |  |  |  |  |
| Disagree. | 1 (0,8) | 3 (2,0) | 5 (4,2) | 2 (2,7) | 0,135 |
| Neutral. | 0 (0,0) | 1 (0,7) | 2 (1,7) | 3 (4,0) |  |
| Agree. | 122 (99,2) | 146 (97,3) | 113 (94,2) | 70 (93,3) |  |
| **2) Breast clipping before NAC is indicated only when BCS is possible?** |  |  |  |  |  |
| Disagree. | 29 (23,6) | 34 (22,7) | 22 (18,3) | 23 (30,7) | 0,342 |
| Neutral. | 1 (0,8) | 4 (2,7) | 3 (2,5) | 0 (0,0) |  |
| Agree. | 93 (75,6) | 112 (74,7) | 95 (79,2) | 52 (69,3) |  |
| **3) In T4b tumors clipping (breast or axilla) before NAC in unnecessary** |  |  |  |  |  |
| Disagree. | 38 (30,9) | 41 (27,3) | 21 (17,5) | 19 (25,3) | 0,334 |
| Neutral. | 5 (4,1) | 4 (2,7) | 4 (3,3) | 3 (4,0) |  |
| Agree. | 80 (65,0) | 105 (70,0) | 95 (79,2) | 53 (70,7) |  |
| **4) Suspicious lymph node clipping is necessary before NAC?** |  |  |  |  |  |
| Disagree. | 83 (67,5) | 98 (65,3) | 67 (55,8) | 37 (49,3) | **0,015** |
| Neutral. | 11 (8,9) | 17 (11,3) | 9 (7,5) | 5 (6,7) |  |
| Agree. | 29 (23,6) | 35 (23,3) | 44 (36,7) | **33 (44,0)≠** |  |
| **5) Positive lymph node clipping is necessary before NAC?** |  |  |  |  |  |
| Disagree. | 64 (52,0) | 86 (57,3) | 57 (47,5) | 26 (34,7) | 0,051 |
| Neutral. | 9 (7,3) | 12 (8,0) | 9 (7,5) | 5 (6,7) |  |
| Agree. | 50 (40,7) | 52 (34,7) | 54 (45,0) | 44 (58,7) |  |
| **6) Which method is available for clipping the breast in your routine?** |  |  |  |  |  |
| None. | 3 (2,4) | 2 (1,3) | 4 (3,3) | 6 (8,0) | **0,012** |
| Coal. | 8 (6,5) | 16 (10,7) | **24 (20,0)≠** | 12 (16,0) |  |
| Metallic Clip. | 109 (88,6) | 126 (84,0) | 99 (82,5) | 55 (73,3) |  |
| Skin tattoo. | 54 (43,9) | 64 (42,7) | 35 (29,2) | 34 (45,3) |  |
| Radioactive Seeds. | 9 (7,3) | 8 (5,3) | 11 (9,2) | 7 (9,3) |  |
| **7) Which method is available for clipping the axilla in your routine?** |  |  |  |  |  |
| None. | 46 (37,4) | 61 (40,7) | 34 (28,3) | 19 (25,3) | 0,051 |
| Coal. | 16 (13,0) | 20 (13,3) | 27 (22,5) | 17 (22,7) |  |
| Metallic Clip. | 66 (53,7) | 76 (50,7) | 68 (56,7) | 38 (50,7) |  |
| Skin tattoo. | 8 (6,5) | 12 (8,0) | 9 (7,5) | 11 (14,7) |  |
| Radioactive Seeds. | 9 (7,3) | 8 (5,3) | 9 (7,5) | 10 (13,3) |  |
| **8) What is your clipping preference?** |  |  |  |  |  |
| None. | 1 (0,8) | 0 (0,0) | 3 (2,5) | 1 (1,3) | **0,002** |
| Coal. | 4 (3,3) | 2 (1,3) | 7 (5,8) | 6 (8,0) |  |
| Metallic Clip. | 88 (71,5) | **115 (76,7)≠** | 81 (67,5) | 42 (56,0) |  |
| Skin tattoo. | 2 (1,6) | 5 (3,3) | 2 (1,7) | **9 (12,0)≠** |  |
| Radioactive Seeds. | 28 (22,8) | 28 (18,7) | 27 (22,5) | 17 (22,7) |  |
| **9) If the clip is visible by US this would be my preference** |  |  |  |  |  |
| Disagree. | 13 (10,6) | 11 (7,3) | 7 (5,8) | 20 (26,7) | **<0,001** |
| Neutral. | 4 (3,3) | 4 (2,7) | 3 (2,5) | 1 (1,3) |  |
| Agree. | 106 (86,2) | **135 (90,0)≠** | **110 (91,7)≠** | 54 (72,0) |  |
| **10) Image or clinical axillary invasion is sufficient?** |  |  |  |  |  |
| Disagree. | 84 (68,3) | 106 (70,7) | 88 (73,3) | 57 (76,0) | 0,075 |
| Neutral. | 2 (1,6) | 0 (0,0) | 5 (4,2) | 0 (0,0) |  |
| Agree. | 37 (30,1) | 44 (29,3) | 27 (22,5) | 18 (24,0) |  |
| **11) For breast/axilla clipping the material and technics are important issues** |  |  |  |  |  |
| Disagree. | 8 (6,5) | 7 (4,7) | 2 (1,7) | 2 (2,7) | 0,323 |
| Neutral. | 2 (1,6) | 3 (2,0) | 3 (2,5) | 4 (5,3) |  |
| Agree. | 113 (91,9) | 140 (93,3) | 115 (95,8) | 69 (92,0) |  |
| **12) Available of breast image specialist is considered to breast clipping** |  |  |  |  |  |
| Disagree. | 6 (4,9) | 12 (8,0) | 5 (4,2) | 3 (4,0) | 0,474 |
| Neutral. | 5 (4,1) | 2 (1,3) | 2 (1,7) | 1 (1,3) |  |
| Agree. | 112 (91,1) | 136 (90,7) | 113 (94,2) | 71 (94,7) |  |
| **13) Available of breast image specialist is considered to axilla clipping** |  |  |  |  |  |
| Disagree. | 20 (16,3) | 24 (16,0) | 10 (8,3) | 8 (10,7) | 0,147 |
| Neutral. | 10 (8,1) | 6 (4,0) | 11 (9,2) | 9 (12,0) |  |
| Agree. | 93 (75,6) | 120 (80,0) | 99 (82,5) | 58 (77,3) |  |
| **14) When is the best moment to clip the breast?** |  |  |  |  |  |
| After pathologic hematoxylin-eosin confirmation of malignancy in the biopsy. | 24 (19,5) | 40 (26,7) | 27 (22,5) | 22 (29,3) | **0,008** |
| After immunohistochemistry prognostic panel result. | **50 (40,7)≠** | 57 (38,0) | 44 (36,7) | 16 (21,3) |  |
| During the NAC according to clinical and imaging response. | 22 (17,9) | 11 (7,3) | 9 (7,5) | 5 (6,7) |  |
| Breast tumor marking is not needed before NAC | 0 (0,0) | 1 (0,7) | 2 (1,7) | 1 (1,3) |  |
| During the biopsy. | 27 (22,0) | 41 (27,3) | 38 (31,7) | **31 (41,3)≠** |  |
| **15) What is your opinion about training in lymph node clipping?** |  |  |  |  |  |
| Dispensable. | 24 (19,5) | 38 (25,3) | 22 (18,3) | 15 (20,0) | 0,271 |
| Optional. | 11 (8,9) | 16 (10,7) | 10 (8,3) | 5 (6,7) |  |
| Neutral. | 20 (16,3) | 15 (10,0) | 15 (12,5) | 6 (8,0) |  |
| Important | 49 (39,8) | 61 (40,7) | 51 (42,5) | 27 (36,0) |  |
| Indispensable | 19 (15,4) | 20 (13,3) | 22 (18,3) | 22 (29,3) |  |
| **16) Have you ever clipped a lymph node?** |  |  |  |  |  |
| Never. | 78 (63,4) | 94 (62,7) | 69 (57,5) | 40 (53,3) | **0,033** |
| 1- 5 procedures. | 29 (23,6) | 31 (20,7) | 30 (25,0) | 14 (18,7) |  |
| 5- 10 procedures | 7 (5,7) | 9 (6,0) | 9 (7,5) | 2 (2,7) |  |
| 10 – 20 procedures | 2 (1,6) | 4 (2,7) | 7 (5,8) | 5 (6,7) |  |
| More than 20 procedures | 7 (5,7) | 12 (8,0) | 5 (4,2) | **14 (18,7)≠** |  |
| **17) The type of neoadjuvant treatment does not interfere with my clipping** |  |  |  |  |  |
| Disagree. | **66 (53,7)≠** | 68 (45,3) | 47 (39,2) | 23 (30,7) | **0,018** |
| Neutral. | 8 (6,5) | 4 (2,7) | 7 (5,8) | 3 (4,0) |  |
| Agree. | 49 (39,8) | 78 (52,0) | 66 (55,0) | **49 (65,3)≠** |  |
| **18) The cancer subtype changes my clipping recommendation** |  |  |  |  |  |
| Disagree. | **63 (51,2)≠** | 56 (37,3) | 33 (27,5) | 29 (38,7) | **0,019** |
| Neutral. | 6 (4,9) | 7 (4,7) | 9 (7,5) | 4 (5,3) |  |
| Agree. | 54 (43,9) | 87 (58,0) | **78 (65,0)≠** | 42 (56,0) |  |
| **19) SNB is possible after cN1 and complete clinical/image response** |  |  |  |  |  |
| Disagree. | 6 (4,9) | 11 (7,3) | 4 (3,3) | 8 (10,7) | 0,383 |
| Neutral. | 3 (2,4) | 1 (0,7) | 2 (1,7) | 1 (1,3) |  |
| Agree. | 114 (92,7) | 138 (92,0) | 114 (95,0) | 66 (88,0) |  |
| **20) SNB is possible after cN2 and complete clinical/image response** |  |  |  |  |  |
| Disagree. | **39 (31,7)≠** | 38 (25,3) | 23 (19,2) | 16 (21,3) | **0,027** |
| Neutral. | 6 (4,9) | 1 (0,7) | 5 (4,2) | 0 (0,0) |  |
| Agree. | 78 (63,4) | 111 (74,0) | 92 (76,7) | 59 (78,7) |  |
| **21) Axillary clipping is not necessary when use both technetium and blue dye** |  |  |  |  |  |
| Disagree. | 33 (26,8) | 40 (26,7) | 25 (20,8) | 27 (36,0) | 0,068 |
| Neutral. | 14 (11,4) | 13 (8,7) | 8 (6,7) | 1 (1,3) |  |
| Agree. | 76 (61,8) | 97 (64,7) | 87 (72,5) | 47 (62,7) |  |
| **22) SNB with double marker should be my choice** |  |  |  |  |  |
| Disagree. | 29 (23,6) | 46 (30,7) | 25 (20,8) | 18 (24,0) | 0,373 |
| Neutral. | 13 (10,6) | 9 (6,0) | 8 (6,7) | 4 (5,3) |  |
| Agree. | 81 (65,9) | 95 (63,3) | 87 (72,5) | 53 (70,7) |  |
| **23) Without double marker axillary clipping is a good option** |  |  |  |  |  |
| Disagree. | 29 (23,6) | 44 (29,3) | 28 (23,3) | 25 (33,3) | 0,644 |
| Neutral. | 13 (10,6) | 18 (12,0) | 14 (11,7) | 6 (8,0) |  |
| Agree. | 81 (65,9) | 88 (58,7) | 78 (65,0) | 44 (58,7) |  |
| **24) Axillary clearance is my preference when only blue dye or technetium is available.** |  |  |  |  |  |
| Disagree. | 90 (73,2) | 130 (86,7) | 90 (75,0) | 53 (70,7) | 0,080 |
| Neutral. | 5 (4,1) | 3 (2,0) | 4 (3,3) | 4 (5,3) |  |
| Agree. | 28 (22,8) | 17 (11,3) | 26 (21,7) | 18 (24,0) |  |
| **25) Axillary clearance is my preference when at least 3 nodes are found** |  |  |  |  |  |
| Disagree. | 64 (52,0) | 85 (56,7) | 63 (52,5) | 37 (49,3) | 0,720 |
| Neutral. | 5 (4,1) | 8 (5,3) | 10 (8,3) | 4 (5,3) |  |
| Agree. | 54 (43,9) | 57 (38,0) | 47 (39,2) | 34 (45,3) |  |
| **26) When the breast is not clipped and a complete response is found I do prefer mastectomy** |  |  |  |  |  |
| Disagree. | 37 (30,1) | 63 (42,0) | 60 (50,0) | **45 (60,0)≠** | **0,002** |
| Neutral. | 3 (2,4) | 4 (2,7) | 4 (3,3) | 3 (4,0) |  |
| Agree. | **83 (67,5)≠** | 83 (55,3) | 56 (46,7) | 27 (36,0) |  |
| **27) Activated coal in the breast do not affect pathological report** |  |  |  |  |  |
| Disagree. | 36 (29,3) | 46 (30,7) | 34 (28,3) | 20 (26,7) | **0,001** |
| Neutral. | **58 (47,2)≠** | **69 (46,0)≠** | 41 (34,2) | 19 (25,3) |  |
| Agree. | 29 (23,6) | 35 (23,3) | 45 (37,5) | **36 (48,0)≠** |  |
| **28) Activated coal in the axilla do not affect pathological report** |  |  |  |  |  |
| Disagree. | 39 (31,7) | 50 (33,3) | 36 (30,0) | 20 (26,7) | **0,005** |
| Neutral. | 53 (43,1) | 63 (42,0) | 38 (31,7) | 19 (25,3) |  |
| Agree. | 31 (25,2) | 37 (24,7) | 46 (38,3) | **36 (48,0)≠** |  |

*Chi-square; ≠Post hoc; n, absolute frequency; %, relative frequency.

Legend: BCS, breast conserving surgery; HE, hematoxylin-eosin; NAC, neoadjuvant chemotherapy; SLNB, sentinel lymph node biopsy; US, ultrasound.

# Table S6. Distribution of variables according to the mastologist's gender group.

|  | Gender | | *p** |
| --- | --- | --- | --- |
|  | Female | Male |  |
| **1) Breast clipping before NAC is indicated?** |  |  |  |
| Disagree. | 7 (3,0) | 4 (1,7) | 0,471 |
| Neutral. | 2 (0,9) | 4 (1,7) |  |
| Agree. | 223 (96,1) | 228 (96,6) |  |
| **2) Breast clipping before NAC is indicated only when BCS is possible?** | | |  |
| Disagree. | 60 (25,9) | 48 (20,3) | 0,308 |
| Neutral. | 3 (1,3) | 5 (2,1) |  |
| Agree. | 169 (72,8) | 183 (77,5) |  |
| **3) In T4b tumors clipping (breast or axilla) before NAC in unnecessary** | | |  |
| Disagree. | 61 (26,3) | 58 (24,6) | 0,586 |
| Neutral. | 6 (2,6) | 10 (4,2) |  |
| Agree. | 165 (71,1) | 168 (71,2) |  |
| **4) Suspicious lymph node clipping is necessary before NAC?** | |  |  |
| Disagree. | 140 (60,3) | 145 (61,4) | 0,580 |
| Neutral. | 24 (10,3) | 18 (7,6) |  |
| Agree. | 68 (29,3) | 73 (30,9) |  |
| **5) Positive lymph node clipping is necessary before NAC?** | |  |  |
| Disagree. | 114 (49,1) | 119 (50,4) | 0,133 |
| Neutral. | 23 (9,9) | 12 (5,1) |  |
| Agree. | 95 (40,9) | 105 (44,5) |  |
| **6) Which method is available for clipping the breast in your routine?** | |  |  |
| None. | 4 (1,7) | 11 (4,7) | **0,024** |
| Coal. | 27 (11,6) | 33 (14,0) |  |
| Metallic Clip. | **202 (87,1)≠** | 187 (79,2) |  |
| Skin tattoo. | 91 (39,2) | 96 (40,7) |  |
| Radioactive Seeds. | 12 (5,2) | 23 (9,7) |  |
| **7) Which method is available for clipping the axilla in your routine?** | |  |  |
| None. | 80 (34,5) | 80 (33,9) | **0,026** |
| Coal. | 38 (16,4) | 42 (17,8) |  |
| Metallic Clip. | 125 (53,9) | 123 (52,1) |  |
| Skin tattoo. | 13 (5,6) | **27 (11,4)≠** |  |
| Radioactive Seeds. | 13 (5,6) | 23 (9,7) |  |
| **8) What is your clipping preference?** |  |  |  |
| None. | 0 (0,0) | 5 (2,1) | **0,001** |
| Coal. | 7 (3,0) | 12 (5,1) |  |
| Metallic Clip. | **174 (75,0)≠** | 152 (64,4) |  |
| Skin tattoo. | 2 (0,9) | **16 (6,8)≠** |  |
| Radioactive Seeds. | 49 (21,1) | 51 (21,6) |  |
| **9) If the clip is visible by US this would be my preference** | |  |  |
| Disagree. | 19 (8,2) | 32 (13,6) | **0,033** |
| Neutral. | 3 (1,3) | 9 (3,8) |  |
| Agree. | **210 (90,5)≠** | 195 (82,6) |  |
| **10) Image or clinical axillary invasion is sufficient?** | |  |  |
| Disagree. | 174 (75,0) | 161 (68,2) | 0,191 |
| Neutral. | 2 (0,9) | 5 (2,1) |  |
| Agree. | 56 (24,1) | 70 (29,7) |  |
| **11) For breast/axilla clipping the material and technics are important issues** | | |  |
| Disagree. | 8 (3,4) | 11 (4,7) | 0,678 |
| Neutral. | 5 (2,2) | 7 (3,0) |  |
| Agree. | 219 (94,4) | 218 (92,4) |  |
| **12) Available of breast image specialist is considered to breast clipping** | | |  |
| Disagree. | 13 (5,6) | 13 (5,5) | 0,829 |
| Neutral. | 4 (1,7) | 6 (2,5) |  |
| Agree. | 215 (92,7) | 217 (91,9) |  |
| **13) Available of breast image specialist is considered to axilla clipping** | | |  |
| Disagree. | 28 (12,1) | 34 (14,4) | 0,156 |
| Neutral. | 13 (5,6) | 23 (9,7) |  |
| Agree. | 191 (82,3) | 179 (75,8) |  |
| **14) When is the best moment to clip the breast?** |  |  |  |
| After pathologic hematoxylin-eosin confirmation of malignancy in the biopsy. | 54 (23,3) | 59 (25,0) | 0,359 |
| After immunohistochemistry prognostic panel result. | 86 (37,1) | 81 (34,3) |  |
| During the NAC according to clinical and imaging response. | 23 (9,9) | 24 (10,2) |  |
| Breast tumor marking is not needed before NAC | 0 (0,0) | 4 (1,7) |  |
| During the biopsy. | 69 (29,7) | 68 (28,8) |  |
| **15) What is your opinion about training in lymph node clipping?** | |  |  |
| Dispensable. | 55 (23,7) | 44 (18,6) | 0,727 |
| Optional. | 26 (11,2) | 30 (12,7) |  |
| Neutral. | 19 (8,2) | 23 (9,7) |  |
| Important | 92 (39,7) | 96 (40,7) |  |
| Indispensable | 40 (17,2) | 43 (18,2) |  |
| **16) Have you ever clipped a lymph node?** |  |  |  |
| Never. | 150 (64,7) | 131 (55,5) | **0,001** |
| 1- 5 procedures. | 55 (23,7) | 49 (20,8) |  |
| 5- 10 procedures | 14 (6,0) | 13 (5,5) |  |
| 10 – 20 procedures | 5 (2,2) | 13 (5,5) |  |
| More than 20 procedures | 8 (3,4) | **30 (12,7)≠** |  |
| **17) The type of neoadjuvant treatment does not interfere with my clipping** | | |  |
| Disagree. | **113 (48,7)≠** | 91 (38,6) | **0,037** |
| Neutral. | 7 (3,0) | 15 (6,4) |  |
| Agree. | 112 (48,3) | 130 (55,1) |  |
| **18) The cancer subtype changes my clipping recommendation** | |  |  |
| Disagree. | 98 (42,2) | 83 (35,2) | 0,198 |
| Neutral. | 10 (4,3) | 16 (6,8) |  |
| Agree. | 124 (53,4) | 137 (58,1) |  |
| **19) SNB is possible after cN1 and complete clinical/image response** | | |  |
| Disagree. | 13 (5,6) | 16 (6,8) | 0,811 |
| Neutral. | 3 (1,3) | 4 (1,7) |  |
| Agree. | 216 (93,1) | 216 (91,5) |  |
| **20) SNB is possible after cN2 and complete clinical/image response** | | |  |
| Disagree. | 65 (28,0) | 51 (21,6) | 0,254 |
| Neutral. | 5 (2,2) | 7 (3,0) |  |
| Agree. | 162 (69,8) | 178 (75,4) |  |
| **21) Axillary clipping is not necessary when use both technetium and blue dye** | | |  |
| Disagree. | 64 (27,6) | 61 (25,8) | 0,153 |
| Neutral. | 23 (9,9) | 13 (5,5) |  |
| Agree. | 145 (62,5) | 162 (68,6) |  |
| **22) SNB with double marker should be my choice** | |  |  |
| Disagree. | 60 (25,9) | 58 (24,6) | 0,854 |
| Neutral. | 18 (7,8) | 16 (6,8) |  |
| Agree. | 154 (66,4) | 162 (68,6) |  |
| **23) Without double marker axillary clipping is a good option** | |  |  |
| Disagree. | 60 (25,9) | 66 (28,0) | 0,773 |
| Neutral. | 24 (10,3) | 27 (11,4) |  |
| Agree. | 148 (63,8) | 143 (60,6) |  |
| **24) Axillary clearance is my preference when only blue dye or technetium is available.** | | | |
| Disagree. | 186 (80,2) | 177 (75,0) | 0,350 |
| Neutral. | 6 (2,6) | 10 (4,2) |  |
| Agree. | 40 (17,2) | 49 (20,8) |  |
| **25) Axillary clearance is my preference when at least 3 nodes are found** | | |  |
| Disagree. | 116 (50,0) | 133 (56,4) | 0,384 |
| Neutral. | 14 (6,0) | 13 (5,5) |  |
| Agree. | 102 (44,0) | 90 (38,1) |  |
| **26) When the breast is not clipped and a complete response is found I do prefer mastectomy** | | | |
| Disagree. | 85 (36,6) | **120 (50,8)≠** | **0,007** |
| Neutral. | 7 (3,0) | 7 (3,0) |  |
| Agree. | **140 (60,3)≠** | 109 (46,2) |  |
| **27) Activated coal in the breast do not affect pathological report** | | |  |
| Disagree. | 72 (31,0) | 64 (27,1) | **0,011** |
| Neutral. | 103 (44,4) | 84 (35,6) |  |
| Agree. | 57 (24,6) | **88 (37,3)≠** |  |
| **28) Activated coal in the axilla do not affect pathological report** | |  |  |
| Disagree. | 77 (33,2) | 68 (28,8) | **0,009** |
| Neutral. | **96 (41,4)≠** | 77 (32,6) |  |
| Agree. | 59 (25,4) | **91 (38,6)≠** |  |

*Chi-square; ≠Post hoc; n, absolute frequency; %, relative frequency.

Legend: BCS, breast conserving surgery; HE, hematoxylin-eosin; NAC, neoadjuvant chemotherapy; SLNB, sentinel lymph node biopsy; US, ultrasound.

# Table S7. Distribution of variables according to the certification board time.

|  | Board certification time | | | *p** |
| --- | --- | --- | --- | --- |
|  | < 5 years | 5 a 20 years | > 20 years |  |
| **1) Breast clipping before NAC is indicated?** | |  |  |  |
| Disagree. | 0 (0,0) | 3 (1,6) | 7 (4,4) | 0,159 |
| Neutral. | 0 (0,0) | 1 (0,5) | 3 (1,9) |  |
| Agree. | 49 (100,0) | 186 (97,9) | 148 (93,7) |  |
| **2) Breast clipping before NAC is indicated only when BCS is possible?** | | | |  |
| Disagree. | 12 (24,5) | 38 (20,0) | 32 (20,3) | 0,638 |
| Neutral. | 1 (2,0) | 5 (2,6) | 1 (0,6) |  |
| Agree. | 36 (73,5) | 147 (77,4) | 125 (79,1) |  |
| **3) In T4b tumors clipping (breast or axilla) before NAC in unnecessary** | | | |  |
| Disagree. | 9 (18,4) | 57 (30,0) | 31 (19,6) | 0,160 |
| Neutral. | 2 (4,1) | 4 (2,1) | 4 (2,5) |  |
| Agree. | 38 (77,6) | 129 (67,9) | 123 (77,8) |  |
| **4) Suspicious lymph node clipping is necessary before NAC?** | | |  |  |
| Disagree. | 28 (57,1) | 126 (66,3) | 90 (57,0) | **0,001** |
| Neutral. | 3 (6,1) | **23 (12,1)≠** | 6 (3,8) |  |
| Agree. | 18 (36,7) | 41 (21,6) | **62 (39,2)≠** |  |
| **5) Positive lymph node clipping is necessary before NAC?** | | |  |  |
| Disagree. | 22 (44,9) | 107 (56,3) | 76 (48,1) | **0,022** |
| Neutral. | 2 (4,1) | 18 (9,5) | 6 (3,8) |  |
| Agree. | **25 (51,0)≠** | 65 (34,2) | **76 (48,1)≠** |  |
| **6) Which method is available for clipping the breast in your routine?** | | |  |  |
| None. | 1 (2,0) | 2 (1,1) | 7 (4,4) | **0,011** |
| Coal. | 5 (10,2) | 16 (8,4) | **30 (19,0)≠** |  |
| Metallic Clip. | 40 (81,6) | 169 (88,9) | 127 (80,4) |  |
| Skin tattoo. | 23 (46,9) | 76 (40,0) | 57 (36,1) |  |
| Radioactive Seeds. | 1 (2,0) | 8 (4,2) | **20 (12,7)≠** |  |
| **7) Which method is available for clipping the axilla in your routine?** | | |  |  |
| None. | **21 (42,9)≠** | 72 (37,9) | 41 (25,9) | **0,023** |
| Coal. | 6 (12,2) | 23 (12,1) | **40 (25,3)≠** |  |
| Metallic Clip. | 25 (51,0) | 100 (52,6) | 87 (55,1) |  |
| Skin tattoo. | 2 (4,1) | 10 (5,3) | 17 (10,8) |  |
| Radioactive Seeds. | 1 (2,0) | 10 (5,3) | 20 (12,7)≠ |  |
| **8) What is your clipping preference?** | |  |  |  |
| None. | 0 (0,0) | 1 (0,5) | 3 (1,9) | **0,021** |
| Coal. | 2 (4,1) | 4 (2,1) | 11 (7,0) |  |
| Metallic Clip. | **41 (83,7)≠** | 141 (74,2) | 97 (61,4) |  |
| Skin tattoo. | 1 (2,0) | 4 (2,1) | 10 (6,3) |  |
| Radioactive Seeds. | 5 (10,2) | 40 (21,1) | 37 (23,4) |  |
| **9) If the clip is visible by US this would be my preference** | | |  |  |
| Disagree. | 3 (6,1) | 16 (8,4) | 24 (15,2) | 0,206 |
| Neutral. | 1 (2,0) | 3 (1,6) | 4 (2,5) |  |
| Agree. | 45 (91,8) | 171 (90,0) | 130 (82,3) |  |
| **10) Image or clinical axillary invasion is sufficient?** | |  |  |  |
| Disagree. | 31 (63,3) | 142 (74,7) | 113 (71,5) | 0,105 |
| Neutral. | 2 (4,1) | 0 (0,0) | 4 (2,5) |  |
| Agree. | 16 (32,7) | 48 (25,3) | 41 (25,9) |  |
| **11) For breast/axilla clipping the material and technics are important issues** | | | |  |
| Disagree. | 1 (2,0) | 10 (5,3) | 5 (3,2) | 0,322 |
| Neutral. | 3 (6,1) | 3 (1,6) | 6 (3,8) |  |
| Agree. | 45 (91,8) | 177 (93,2) | 147 (93,0) |  |
| **12) Available of breast image specialist is considered to breast clipping** | | | |  |
| Disagree. | 1 (2,0) | 12 (6,3) | 9 (5,7) | 0,380 |
| Neutral. | 3 (6,1) | 4 (2,1) | 3 (1,9) |  |
| Agree. | 45 (91,8) | 174 (91,6) | 146 (92,4) |  |
| **13) Available of breast image specialist is considered to axilla clipping** | | | |  |
| Disagree. | 8 (16,3) | 29 (15,3) | 18 (11,4) | 0,529 |
| Neutral. | 2 (4,1) | 12 (6,3) | 15 (9,5) |  |
| Agree. | 39 (79,6) | 149 (78,4) | 125 (79,1) |  |
| **14) When is the best moment to clip the breast?** | |  |  |  |
| After pathologic hematoxylin-eosin confirmation of malignancy in the biopsy. | 9 (18,4) | 40 (21,1) | 40 (25,3) | 0,123 |
| After immunohistochemistry prognostic panel result. | 17 (34,7) | 79 (41,6) | 51 (32,3) |  |
| During the NAC according to clinical and imaging response. | 9 (18,4) | 19 (10,0) | 10 (6,3) |  |
| Breast tumor marking is not needed before NAC | 0 (0,0) | 1 (0,5) | 3 (1,9) |  |
| During the biopsy. | 14 (28,6) | 51 (26,8) | 54 (34,2) |  |
| **15) What is your opinion about training in lymph node clipping?** | | |  |  |
| Dispensable. | 11 (22,4) | 46 (24,2) | 32 (20,3) | 0,392 |
| Optional. | 5 (10,2) | 25 (13,2) | 18 (11,4) |  |
| Neutral. | 4 (8,2) | 16 (8,4) | 15 (9,5) |  |
| Important | 19 (38,8) | 83 (43,7) | 60 (38,0) |  |
| Indispensable | 10 (20,4) | 20 (10,5) | 33 (20,9) |  |
| **16) Have you ever clipped a lymph node?** | |  |  |  |
| Never. | 31 (63,3) | 119 (62,6) | 85 (53,8) | 0,221 |
| 1- 5 procedures. | 12 (24,5) | 43 (22,6) | 38 (24,1) |  |
| 5- 10 procedures | 5 (10,2) | 10 (5,3) | 10 (6,3) |  |
| 10 – 20 procedures | 1 (2,0) | 5 (2,6) | 8 (5,1) |  |
| More than 20 procedures | 0 (0,0) | 13 (6,8) | 17 (10,8) |  |
| **17) The type of neoadjuvant treatment does not interfere with my clipping** | | | |  |
| Disagree. | 25 (51,0) | 89 (46,8) | 54 (34,2) | **0,005** |
| Neutral. | 6 (12,2) | 6 (3,2) | 9 (5,7) |  |
| Agree. | 18 (36,7) | 95 (50,0) | 95 (60,1) |  |
| **18) The cancer subtype changes my clipping recommendation** | | |  |  |
| Disagree. | 24 (49,0) | 77 (40,5) | 54 (34,2) | 0,103 |
| Neutral. | 4 (8,2) | 6 (3,2) | 12 (7,6) |  |
| Agree. | 21 (42,9) | 107 (56,3) | 92 (58,2) |  |
| **19) SNB is possible after cN1 and complete clinical/image response** | | |  |  |
| Disagree. | 4 (8,2) | 12 (6,3) | 8 (5,1) | 0,791 |
| Neutral. | 0 (0,0) | 1 (0,5) | 2 (1,3) |  |
| Agree. | 45 (91,8) | 177 (93,2) | 148 (93,7) |  |
| **20) SNB is possible after cN2 and complete clinical/image response** | | |  |  |
| Disagree. | **18 (36,7)≠** | 54 (28,4) | 29 (18,4) | **0,030** |
| Neutral. | 2 (4,1) | 2 (1,1) | 3 (1,9) |  |
| Agree. | 29 (59,2) | 134 (70,5) | **126 (79,7)≠** |  |
| **21) Axillary clipping is not necessary when use both technetium and blue dye** | | | |  |
| Disagree. | 11 (22,4) | 46 (24,2) | 43 (27,2) | 0,610 |
| Neutral. | 6 (12,2) | 15 (7,9) | 9 (5,7) |  |
| Agree. | 32 (65,3) | 129 (67,9) | 106 (67,1) |  |
| **22) SNB with double marker should be my choice** | |  |  |  |
| Disagree. | 11 (22,4) | 57 (30,0) | 35 (22,2) | 0,516 |
| Neutral. | 3 (6,1) | 12 (6,3) | 12 (7,6) |  |
| Agree. | 35 (71,4) | 121 (63,7) | 111 (70,3) |  |
| **23) Without double marker axillary clipping is a good option** | | |  |  |
| Disagree. | 12 (24,5) | 49 (25,8) | 46 (29,1) | 0,926 |
| Neutral. | 5 (10,2) | 20 (10,5) | 18 (11,4) |  |
| Agree. | 32 (65,3) | 121 (63,7) | 94 (59,5) |  |
| **24) Axillary clearance is my preference when only blue dye or technetium is available.** | | | |  |
| Disagree. | 37 (75,5) | **163 (85,8)≠** | 117 (74,1) | **0,037** |
| Neutral. | 3 (6,1) | 2 (1,1) | 7 (4,4) |  |
| Agree. | 9 (18,4) | 25 (13,2) | 34 (21,5) |  |
| **25) Axillary clearance is my preference when at least 3 nodes are found** | | | |  |
| Disagree. | 20 (40,8) | 103 (54,2) | 93 (58,9) | 0,098 |
| Neutral. | 2 (4,1) | 8 (4,2) | 11 (7,0) |  |
| Agree. | 27 (55,1) | 79 (41,6) | 54 (34,2) |  |
| **26) When the breast is not clipped and a complete response is found I do prefer mastectomy** | | | | |
| Disagree. | 18 (36,7) | 69 (36,3) | **86 (54,4)≠** | **0,010** |
| Neutral. | 1 (2,0) | 4 (2,1) | 4 (2,5) |  |
| Agree. | **30 (61,2)≠** | **117 (61,6)≠** | 68 (43,0) |  |
| **27) Activated coal in the breast do not affect pathological report** | | |  |  |
| Disagree. | 16 (32,7) | 53 (27,9) | 49 (31,0) | **0,001** |
| Neutral. | **22 (44,9)≠** | **89 (46,8)≠** | 43 (27,2) |  |
| Agree. | 11 (22,4) | 48 (25,3) | **66 (41,8)≠** |  |
| **28) Activated coal in the axilla do not affect pathological report** | | |  |  |
| Disagree. | 17 (34,7) | 61 (32,1) | 49 (31,0) | **0,004** |
| Neutral. | 19 (38,8) | **80 (42,1)≠** | 41 (25,9) |  |
| Agree. | 13 (26,5) | 49 (25,8) | **68 (43,0)≠** |  |

* Chi-square; ≠Post hoc; n, absolute frequency; %, relative frequency.

Legend: BCS, breast conserving surgery; HE, hematoxylin-eosin; NAC, neoadjuvant chemotherapy; SLNB, sentinel lymph node biopsy; US, ultrasound.

# Table S8. Distribution of variables according to the presence of board certification.

|  | Board certification | | *p** |
| --- | --- | --- | --- |
|  | No | Yes |  |
| **1) Breast clipping before NAC is indicated?** |  |  |  |
| Totally disagree. | 0 (0,0) | 2 (0,5) | 0,653 |
| Partially disagree. | 1 (1,4) | 8 (2,0) |  |
| Neutral. | 2 (2,8) | 4 (1,0) |  |
| Partially agree. | 29 (40,8) | 147 (37,0) |  |
| Totally agree. | 39 (54,9) | 236 (59,4) |  |
| **2) Breast clipping before NAC is indicated only when BCS is possible?** |  |  |  |
| Totally disagree. | 13 (18,3) | 43 (10,8) | 0,055 |
| Partially disagree. | 13 (18,3) | 39 (9,8) |  |
| Neutral. | 1 (1,4) | 7 (1,8) |  |
| Partially agree. | 14 (19,7) | 117 (29,5) |  |
| Totally agree. | 30 (42,3) | 191 (48,1) |  |
| **3) In T4b tumors clipping (breast or axilla) before NAC in unnecessary** |  |  |  |
| Totally disagree. | 7 (9,9) | 32 (8,1) | 0,073 |
| Partially disagree. | 15 (21,1) | 65 (16,4) |  |
| Neutral. | 6 (8,5) | 10 (2,5) |  |
| Partially agree. | 20 (28,2) | 131 (33,0) |  |
| Totally agree. | 23 (32,4) | 159 (40,1) |  |
| **4) Suspicious lymph node clipping is necessary before NAC?** |  |  |  |
| Totally disagree. | 25 (35,2) | 164 (41,3) | 0,344 |
| Partially disagree. | 16 (22,5) | 80 (20,2) |  |
| Neutral. | 10 (14,1) | 32 (8,1) |  |
| Partially agree. | 13 (18,3) | 93 (23,4) |  |
| Totally agree. | 7 (9,9) | 28 (7,1) |  |
| **5) Positive lymph node clipping is necessary before NAC?** |  |  |  |
| Totally disagree. | 14 (19,7) | **129 (32,5)≠** | **0,039** |
| Partially disagree. | 14 (19,7) | 76 (19,1) |  |
| Neutral. | 9 (12,7) | 26 (6,5) |  |
| Partially agree. | 15 (21,1) | 100 (25,2) |  |
| Totally agree. | **19 (26,8)≠** | 66 (16,6) |  |
| **6) Which method is available for clipping the breast in your routine?** |  |  |  |
| None. | 5 (7,0) | 10 (2,5) | **0,041** |
| Coal. | 9 (12,7) | 51 (12,8) |  |
| Metallic clip. | 53 (74,6) | **336 (84,6)≠** |  |
| Skin tattoo. | 31 (43,7) | 156 (39,3) |  |
| Radioactive seeds. | 6 (8,5) | 29 (7,3) |  |
| **7) Which method is available for clipping the axilla in your routine?** |  |  |  |
| None. | 26 (36,6) | 134 (33,8) | **0,039** |
| Coal. | 11 (15,5) | 69 (17,4) |  |
| Metallic clip. | 36 (50,7) | 212 (53,4) |  |
| Skin tattoo. | **11 (15,5)≠** | 29 (7,3) |  |
| Radioactive seeds. | 5 (7,0) | 31 (7,8) |  |
| **8) What is your clipping preference?** |  |  |  |
| Coal. | 2 (2,8) | 17 (4,3) | 0,877 |
| Metallic clip. | 47 (66,2) | 279 (70,3) |  |
| None. | 1 (1,4) | 4 (1,0) |  |
| Skin tattoo. | 3 (4,2) | 15 (3,8) |  |
| Radioactive seeds. | 18 (25,4) | 82 (20,7) |  |
| **9) If the clip is visible by US this would be my preference** |  |  |  |
| Totally disagree. | 4 (5,6) | 23 (5,8) | 0,193 |
| Partially disagree. | 4 (5,6) | 20 (5,0) |  |
| Neutral. | 4 (5,6) | 8 (2,0) |  |
| Partially agree. | 19 (26,8) | 76 (19,1) |  |
| Totally agree. | 40 (56,3) | 270 (68,0) |  |
| **10) Image or clinical axillary invasion is sufficient?** |  |  |  |
| Totally disagree. | 26 (36,6) | 181 (45,6) | 0,591 |
| Partially disagree. | 23 (32,4) | 105 (26,4) |  |
| Neutral. | 1 (1,4) | 6 (1,5) |  |
| Partially agree. | 17 (23,9) | 89 (22,4) |  |
| Totally agree. | 4 (5,6) | 16 (4,0) |  |
| **11) For breast/axilla clipping the material and technics are important issues** |  |  |  |
| Totally disagree. | 2 (2,8) | 7 (1,8) | 0,671 |
| Partially disagree. | 1 (1,4) | 9 (2,3) |  |
| Neutral. | 0 (0,0) | 12 (3,0) |  |
| Partially agree. | 19 (26,8) | 108 (27,2) |  |
| Totally agree. | 49 (69,0) | 261 (65,7) |  |
| **12) Available of breast image specialist is considered to breast clipping** |  |  |  |
| Totally disagree. | 3 (4,2) | 14 (3,5) | 0,679 |
| Partially disagree. | 1 (1,4) | 8 (2,0) |  |
| Neutral. | 0 (0,0) | 10 (2,5) |  |
| Partially agree. | 18 (25,4) | 87 (21,9) |  |
| Totally agree. | 49 (69,0) | 278 (70,0) |  |
| **13) Available of breast image specialist is considered to axilla clipping** |  |  |  |
| Totally disagree. | 4 (5,6) | 39 (9,8) | 0,240 |
| Partially disagree. | 3 (4,2) | 16 (4,0) |  |
| Neutral. | 7 (9,9) | 29 (7,3) |  |
| Partially agree. | 20 (28,2) | 72 (18,1) |  |
| Totally agree. | 37 (52,1) | 241 (60,7) |  |
| **14) When is the best moment to clip the breast?** |  |  |  |
| After pathologic hematoxylin-eosin confirmation of malignancy in the biopsy. | 24 (33,8) | 89 (22,4) | 0,174 |
| After immunohistochemistry prognostic panel result. | 20 (28,2) | 147 (37,0) |  |
| During the NAC according to clinical and imaging response. | 9 (12,7) | 38 (9,6) |  |
| Breast tumor marking is not needed before NAC | 0 (0,0) | 4 (1,0) |  |
| During the biopsy. | 18 (25,4) | 119 (30,0) |  |
| **15) What is your opinion about training in lymph node clipping?** |  |  |  |
| Dispensable. | 10 (14,1) | 89 (22,4) | 0,112 |
| Optional. | 26 (36,6) | 162 (40,8) |  |
| Neutral. | 20 (28,2) | 63 (15,9) |  |
| Important | 7 (9,9) | 35 (8,8) |  |
| Indispensable | 8 (11,3) | 48 (12,1) |  |
| **16) Have you ever clipped a lymph node?** |  |  |  |
| Never. | 11 (15,5) | 93 (23,4) | 0,287 |
| 1- 5 procedures. | 4 (5,6) | 14 (3,5) |  |
| 5- 10 procedures | 2 (2,8) | 25 (6,3) |  |
| 10 – 20 procedures | 8 (11,3) | 30 (7,6) |  |
| More than 20 procedures | 46 (64,8) | 235 (59,2) |  |
| **17) The type of neoadjuvant treatment does not interfere with my clipping** |  |  |  |
| Totally disagree. | 22 (31,0) | 99 (24,9) | 0,107 |
| Partially disagree. | 14 (19,7) | 69 (17,4) |  |
| Neutral. | 1 (1,4) | 21 (5,3) |  |
| Partially agree. | 22 (31,0) | 93 (23,4) |  |
| Totally agree. | 12 (16,9) | 115 (29,0) |  |
| **18) The cancer subtype changes my clipping recommendation** |  |  |  |
| Totally disagree. | 12 (16,9) | 102 (25,7) | 0,263 |
| Partially disagree. | 14 (19,7) | 53 (13,4) |  |
| Neutral. | 4 (5,6) | 22 (5,5) |  |
| Partially agree. | 27 (38,0) | 122 (30,7) |  |
| Totally agree. | 14 (19,7) | 98 (24,7) |  |
| **19) SLNB is possible after cN1 and complete clinical/image response** |  |  |  |
| Totally disagree. | 2 (2,8) | 13 (3,3) | **0,007** |
| Partially disagree. | 3 (4,2) | 11 (2,8) |  |
| Neutral. | 4 (5,6) | 3 (0,8) |  |
| Partially agree. | 16 (22,5) | 57 (14,4) |  |
| Totally agree. | 46 (64,8) | **313 (78,8)≠** |  |
| **20) SLNB is possible after cN2 and complete clinical/image response** |  |  |  |
| Totally disagree. | 5 (7,0) | 58 (14,6) | **0,005** |
| Partially disagree. | 10 (14,1) | 43 (10,8) |  |
| Neutral. | 5 (7,0) | 7 (1,8) |  |
| Partially agree. | 15 (21,1) | **135 (34,0)≠** |  |
| Totally agree. | 36 (50,7) | 154 (38,8) |  |
| **21) Axillary clipping is not necessary when use both technetium and blue dye** |  |  |  |
| Totally disagree. | 9 (12,7) | 43 (10,8) | 0,228 |
| Partially disagree. | 16 (22,5) | 57 (14,4) |  |
| Neutral. | 6 (8,5) | 30 (7,6) |  |
| Partially agree. | 21 (29,6) | 109 (27,5) |  |
| Totally agree. | 19 (26,8) | 158 (39,8) |  |
| **22) SNB with double marker should be my choice** |  |  |  |
| Totally disagree. | 8 (11,3) | 62 (15,6) | 0,809 |
| Partially disagree. | 7 (9,9) | 41 (10,3) |  |
| Neutral. | 7 (9,9) | 27 (6,8) |  |
| Partially agree. | 20 (28,2) | 112 (28,2) |  |
| Totally agree. | 29 (40,8) | 155 (39,0) |  |
| **23) Without double marker axillary clipping is a good option** |  |  |  |
| Totally disagree. | 12 (16,9) | 58 (14,6) | 0,841 |
| Partially disagree. | 7 (9,9) | 49 (12,3) |  |
| Neutral. | 8 (11,3) | 43 (10,8) |  |
| Partially agree. | 26 (36,6) | 164 (41,3) |  |
| Totally agree. | 18 (25,4) | 83 (20,9) |  |
| **24) Axillary clearances is my preference when only blue dye or technetium is available** |  |  |  |
| Totally disagree. | 32 (45,1) | 202 (50,9) | **0,011** |
| Partially disagree. | 14 (19,7) | 115 (29,0) |  |
| Neutral. | 4 (5,6) | 12 (3,0) |  |
| Partially agree. | **18 (25,4)≠** | 44 (11,1) |  |
| Totally agree. | 3 (4,2) | 24 (6,0) |  |
| **25) Axillary clearance is my preference when at least 3 nodes are found** |  |  |  |
| Totally disagree. | 18 (25,4) | 100 (25,2) | 0,556 |
| Partially disagree. | 15 (21,1) | 116 (29,2) |  |
| Neutral. | 6 (8,5) | 21 (5,3) |  |
| Partially agree. | 16 (22,5) | 87 (21,9) |  |
| Totally agree. | 16 (22,5) | 73 (18,4) |  |
| **26) When the breast is not clipped and a complete response is found I do prefer mastectomy** |  |  |  |
| Totally disagree. | 12 (16,9) | 59 (14,9) | 0,177 |
| Partially disagree. | 20 (28,2) | 114 (28,7) |  |
| Neutral. | 5 (7,0) | 9 (2,3) |  |
| Partially agree. | 18 (25,4) | 135 (34,0) |  |
| Totally agree. | 16 (22,5) | 80 (20,2) |  |
| **27) Activated coal in the breast does not affect pathological report** |  |  |  |
| Totally disagree. | 4 (5,6) | 34 (8,6) | 0,507 |
| Partially disagree. | 14 (19,7) | 84 (21,2) |  |
| Neutral. | 33 (46,5) | 154 (38,8) |  |
| Partially agree. | 7 (9,9) | 63 (15,9) |  |
| Totally agree. | 13 (18,3) | 62 (15,6) |  |
| **28) Activated coal in the axilla does not affect pathological report** |  |  |  |
| Totally disagree. | 6 (8,5) | 40 (10,1) | 0,407 |
| Partially disagree. | 12 (16,9) | 87 (21,9) |  |
| Neutral. | 33 (46,5) | 140 (35,3) |  |
| Partially agree. | 9 (12,7) | 72 (18,1) |  |
| Totally agree. | 11 (15,5) | 58 (14,6) |  |

*Chi-square; ≠Post hoc; n, absolute frequency; %, relative frequency.

Legend: BCS, breast conserving surgery; HE, hematoxylin-eosin; NAC, neoadjuvant chemotherapy; SLNB, sentinel lymph node biopsy; US, ultrasound.
